# The world’s most venomous spider is a species complex: systematics of the Sydney funnel-web spider (Atracidae: *Atrax robustus*)

**DOI:** 10.1186/s12862-024-02332-0

**Published:** 2025-01-13

**Authors:** Stephanie F. Loria, Svea-Celina Frank, Nadine Dupérré, Helen M. Smith, Braxton Jones, Bruno A. Buzatto, Danilo Harms

**Affiliations:** 1 Museum of Nature - Hamburg, Leibniz Institute for the Analysis of Biodiversity Change, Hamburg, Germany; 2 Fachbereich Biologie, Universität Hamburg, Hamburg, Germany; 3 Australian Museum, Sydney, Australia; 4 School of Life and Environmental Sciences, The University of Sydney, Sydney, Australia; 5 College of Science and Engineering, Flinders University, Bedford Park, Australia; 6 Research Adjunct, Western Australian Museum, Welshpool, Australia; 7 Honorary Research Fellow, Harry Butler Institute, Murdoch University, Murdoch, Australia

**Keywords:** Antivenoms, Biodiversity, Biogeography, Mygalomorph spiders, Systematics, Taxonomy

## Abstract

The Sydney funnel-web spider *Atrax robustus* O. Pickard-Cambridge, 1877 is an iconic Australian species and considered among the most dangerously venomous spiders for humans. Originally described in 1877 from a single specimen collected in “New Holland”, this spider has a complex taxonomic history. The most recent morphological revision of funnel-web spiders (Atracidae) lists this species as both widespread and common in the Sydney Basin bioregion and beyond, roughly 250 km from the Newcastle area south to the Illawarra, and extending inland across the Blue Mountains. Morphological variability and venom diversity in this species appear to be unusually high, raising questions about species concepts and diversity in these spiders. In this study, we use a combination of molecular phylogenetics, divergence time analyses and morphology to establish the Sydney funnel-web spider as a complex of three species. The “real” Sydney funnel-web spider *Atrax robustus* is relatively widespread in the Sydney metropolitan region. A second species, *Atrax montanus* (Rainbow, 1914), which is revalidated here, overlaps but mainly occurs further south and west, and a third larger species, *Atrax christenseni* sp. nov., is found in a small area surrounding Newcastle to the north. The revised taxonomy for funnel-web spiders may have practical implications for antivenom production and biochemical studies on spider venoms. Although no human fatalities have occurred since the development of antivenom in the 1980s, antivenom for Sydney funnel-web spiders might be optimized by considering biological differentiation at the species level.

**Supplementary Information:**

The online version contains supplementary material available at 10.1186/s12862-024-02332-0.

## Introduction

**Fig. 1:**
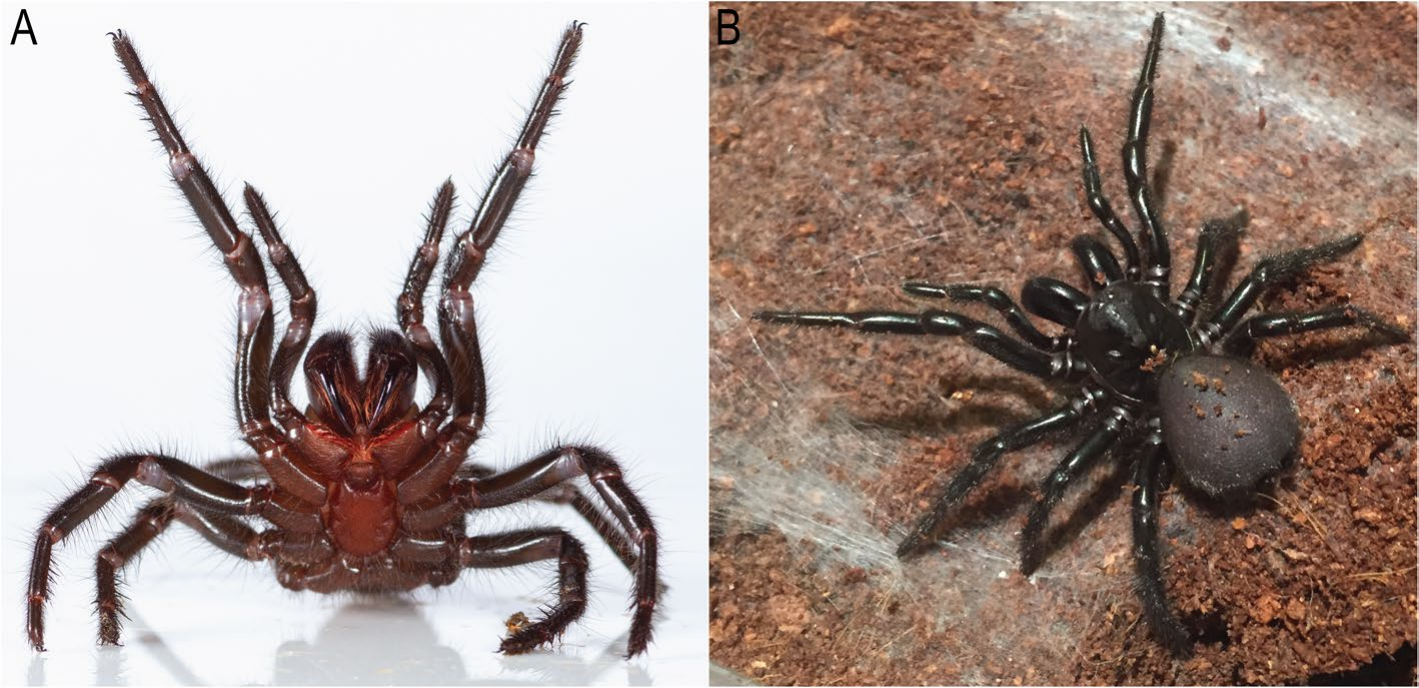
Live photos of female *Atrax robustus* O. Pickard-Cambridge, 1877, West Pennant Hills, New South Wales, Australia, in rearing position (**A**) and dorsal view (**B**) (photos B. Jones)

The Sydney funnel-web spider *Atrax robustus* O. Pickard-Cambridge, 1877 in the family Atracidae Hogg, 1901 is arguably one of the most iconic Australian invertebrates [1–4]. These dark, glossy and large (~ 2–4 cm in body length) spiders (Fig. 1) are considered among the most dangerously venomous spiders for humans, with several deaths resulting from bites before the development of antivenom in the 1980s. This spider has also been a primary target of biochemical research on venom structure and components, including antivenom production. In fact, almost all publications on *Atrax robustus* deal with venom properties (e.g., [5–8]), whereas only few studies have focused on its natural history and taxonomy. The latter is a complex story because this spider was originally described from a single female specimen collected from “New Holland” (today Australia) in the 19th century [9], whereas the male was originally described under a different name from northern Sydney [10]. Both sexes were eventually matched some decades later by Musgrave [11], who was also the first to associate this spider with the death of a child in the northern Sydney suburbs, commenting on the aggressive nature of wild-caught specimens and the effects of their venom based on patient hospital records. Musgrave’s [11] observations on toxicity were confirmed by countless biomedical studies and it is now known that only male spiders of *A. robustus* produce venom peptides that cause serious effects in humans [5–8, 12]. This is unfortunate since male spiders mature in summer and abandon their burrows, usually after rainy periods, to roam in search of mates, often bringing them into contact with humans [13].

**Fig. 2:**
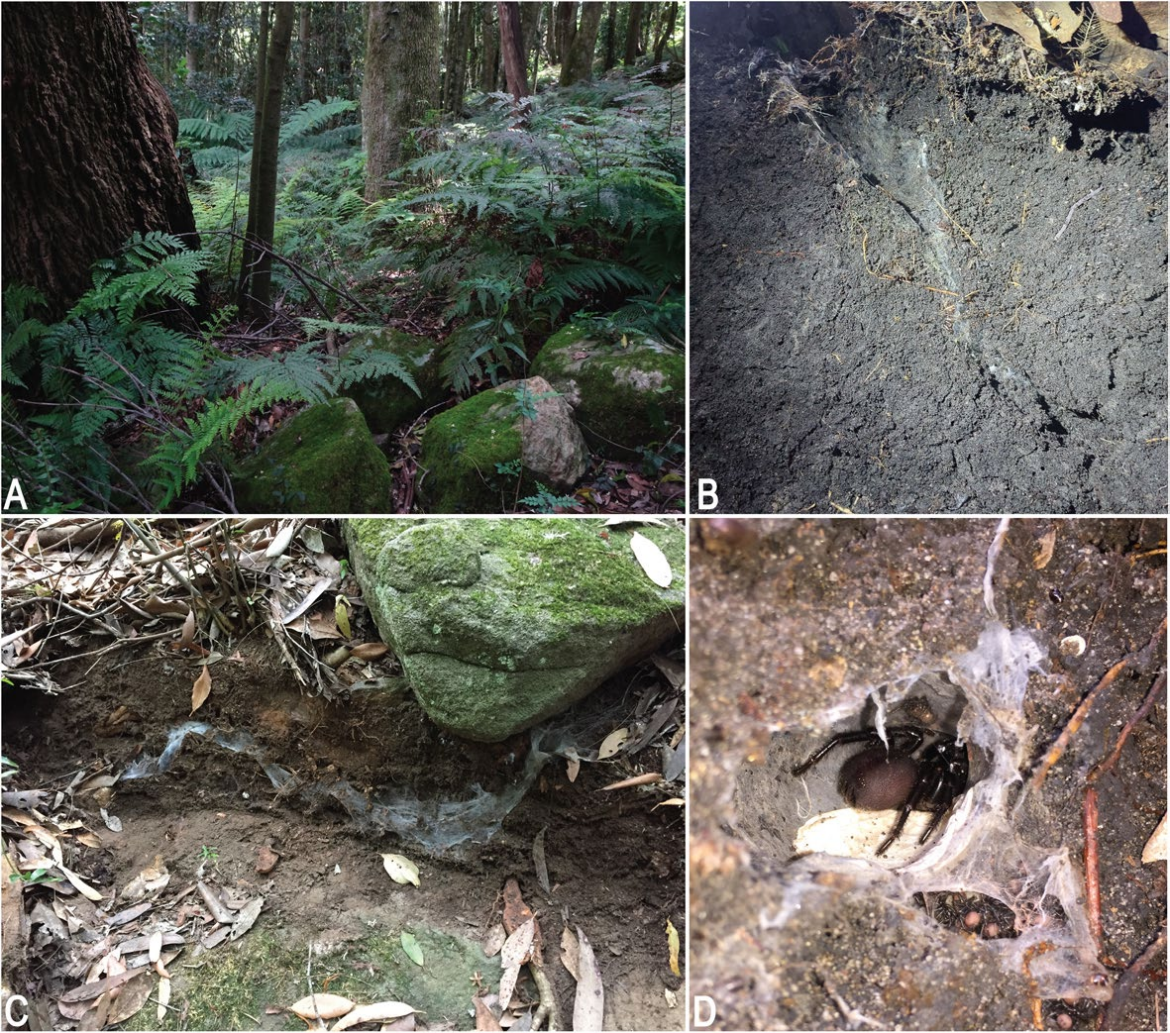
Habitat of *Atrax* O. Pickard-Cambridge, 1877 species. **A.** *Atrax montanus* (Rainbow, 1914), forest habitat in Blue Mountains. **B**. *Atrax christenseni* sp. nov., near Newcastle, burrow under rock. **C**, **D**. *Atrax robustus* O. Pickard-Cambridge, 1877, burrow under rock (**C**), and female with spiderlings (**D**). Photo credit: (**A**) H. Smith/Australian Museum, Sydney; (**B**, **C**) D. Harms; (**D**) B. Jones

**Fig. 3:**
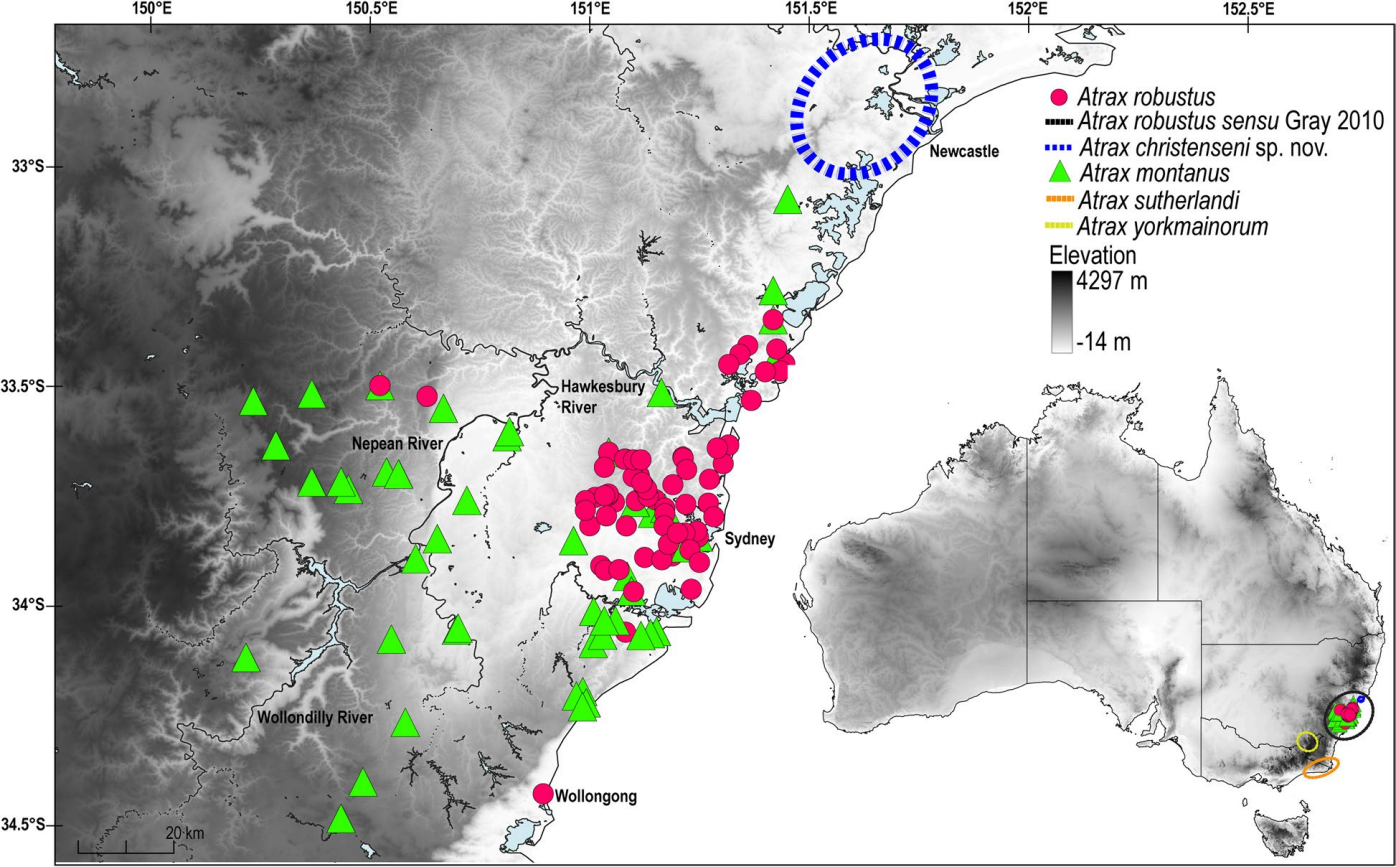
Distribution of *Atrax* O. Pickard-Cambridge, 1877. Records of *Atrax robustus* O. Pickard-Cambridge, 1877, *Atrax montanus* (Rainbow, 1914) and *Atrax christenseni* sp. nov. based on examined material in the present study; ranges for *Atrax yorkmainorum* Gray, 2010 and *Atrax sutherlandi* Gray, 2010 and *Atrax robustus* sensu Gray, 2010 in Australia following Gray [17]. Distribution maps were created using QGIS v.3.14.16 (https://www.qgis.org) by superimposing locality records on elevation data ([23]: GMTED2010; https://www.usgs.gov/coastal-changes-and-impacts/gmted2010)*;* lakes and rivers from Geoscience Australia [24]; and borders and coastlines from Natural Earth (https://www.naturalearthdata.com)

Many more species of funnel-web spiders have been described over the years since Musgrave [11]. Main [14] listed six species of *Atrax* O. Pickard-Cambridge, 1877 from eastern Australia but updated the number to eleven species only two decades later [15]. Main’s [15] latter catalog included *Atrax montanus* (Rainbow, 1914), which was synonymised with *A. robustus* by Gray [16], and several other species that were eventually transferred to its sister genus *Hadronyche* L. Koch, 1873. Main [14] not only reiterated the venomous nature of this species, but also provided a description of its ‘funnel’ webs and natural habitats (Fig. 2). The most comprehensive taxonomic analysis of funnel-webs in Australia was published by Gray [17], who recognized three genera of Australian funnel-web spiders based on somatic and genital morphology, namely *Atrax* for three species from New South Wales, the Australian Capital Territory and Victoria; *Hadronyche* for 31 species from eastern Australia, South Australia and Tasmania (although another species has since been described, bringing the total number of *Hadronyche* species to 32) [18]; and the monotypic *Illawarra* Gray, 2010 from the Illawarra region of New South Wales [18]. These three genera differ in male morphology, with *Atrax* males bearing a prominent conical spur on the tibia of leg II and a flat cephalic region in lateral view. *Hadronyche* males lack the prominent conical spur and have a raised cephalic region, and *Illawarra* males lack the spur and have a flat cephalic region [17]. Within *Atrax*, Gray [17] defined three species primarily based on characteristics of the male embolus, the tip of the copulatory organ, which is used for sperm transfer, and spination features of the male palpal tibia. *Atrax robustus* sensu Gray, 2010 is considered a widespread but morphologically variable species with a linear range of ca. 250 km along the coastline and stretching inland past the Blue Mountains; *Atrax yorkmainorum* Gray, 2010 is a subalpine species that is widely distributed in the high plains of the Australian Alps (New South Wales, Australian Capital Territory); and *Atrax sutherlandi* Gray, 2010 is distributed in temperate forests in southern New South Wales and northern Victoria (Fig. 3). Gray [17] alluded to the preliminary nature of his species concepts noting that *A. robustus* “*specimens from the Hunter Valley/Newcastle region are typically larger than other individuals*”. A subsequent genetic study on *A. sutherlandi* also noted substantial genetic variation between spatially isolated populations, perhaps indicating speciation in the latter [19]. No taxonomic studies at the species level have been published since 2011, although recent phylogenomic studies have classified all Australian funnel-web spiders in their own family (Atracidae Hogg, 1901) and in a sister-relationship with Actinopodidae Simon, 1892 (distributed in Australia and South America) whose males also produce potent venoms. These two families are considered the “venom clade” of mygalomorph spiders [20–22].

Since Gray [17], the original concept of *A. robustus* as a widespread species in the Sydney Basin and beyond was never challenged. The need to review the species concept for *A. robustus* came, in part, from anecdotal evidence of high venom variation and unusually large males that were delivered to the Australian Museum in Sydney and the Australian Reptile Park in Somersby. Antivenom is typically produced from male Sydney funnel-web spiders that are collected during the breeding season by amateur collectors and donated to the Australian Reptile Park, where venoms are extracted. Some of these males were later deposited in the Australian Museum in Sydney, which holds the largest historical collection of funnel-web spiders in the world. Using these collections, newly collected specimens, and Gray’s [17] revision as a baseline, we aimed to: (i) re-assess the taxonomy of *A. robustus* across its distribution via molecular phylogenetic and morphological analyses; (ii) establish the taxonomic status of unusually large-bodied *Atrax* populations near Newcastle [17]; and (iii) identify other species of *Atrax* that may have been previously confused with *A. robustus*.

## Materials and methods

### Material examined

Material examined in the present study is deposited in the following natural history collections: the Australian Museum, Sydney, Australia (AMS); Museum of Nature Hamburg – Zoology (ZMH), Leibniz Institute for the Analysis of Biodiversity Change, Hamburg, Germany; and the Natural History Museum, London, U.K. (BMNH). Specimens used only for morphological analysis include: the types of *Atrax robustus*, *Euctimena tibialis* Rainbow, 1914 and *Poikilomorphia montana* Rainbow, 1914; a male of *A. yorkmainorum* (ZMH-A0024106) from Bago, New South Wales; two females (ZMH-A0016609, ZMH-A0024181) of *A. sutherlandi* from the type locality (Bermagui, southern New South Wales); and 59 males and 65 females of *A. robustus* sensu Gray, 2010 from across this species’ distribution. Specimens used for DNA analysis were acquired from the Australian Reptile Park, Somersby, New South Wales via several private collectors (later deposited in both the AMS and ZMH), and through a 2020 field survey in the Sydney Basin bioregion and Newcastle under permit number SL102329 (Department of Planning, Industry and Environment, New South Wales Government). Juvenile specimens were preserved directly in 100% ethanol and stored at −80 °C in ultra-freezers for tissue preservation; for adult specimens, the third left leg was removed and transferred to 100% ethanol, whereas the body was preserved in 75% ethanol and used for morphological examination.

### Sequencing

Genomic DNA was extracted from 57 *Atrax* and 8 *Hadronyche* specimens using the E.Z.N.A. Tissue DNA kit (Omega Bio-tek, Norcross, GA, U.S.A.), following the manufacturer’s protocol. Three molecular markers were optimized for Sanger sequencing including: a fragment of the mitochondrial cytochrome *c* oxidase subunit I (COI) gene (primers LCO-1490: [25], and HCOoutout: [26]) to resolve shallow nodes, the mitochondrial ribosomal 16S gene (primers 16Sbr and 16Sar: [27]) for less shallow nodes, and a fragment of the nuclear ribosomal 28S gene (28Sa: [28], and LSUR: [29]) to resolve deeper nodes. PCR cleanup was performed using Exonuclease 1 and FastAP (Thermo Fischer Scientific, Waltham, MA, U.S.A.), and uni-directional sequencing was done through Macrogen Europe B.V. (Amsterdam, The Netherlands). Sequences were checked for contamination using BLAST (https://blast.ncbi.nlm.nih.gov) on the NCBI server, and then edited according to IUPAC codes and checked for stop codons in Geneious v.2020.2.4 (Biomatters Limited, Auckland, New Zealand). All sequences generated in this study are deposited on Genbank (https://www.ncbi.nlm.nih.gov/genbank; Table S1); additional outgroup sequences were also downloaded from Genbank (Table S1).

### Phylogenetic analyses

A molecular matrix was compiled to perform phylogenetic analysis of *Atrax robustus* sensu Gray, 2010. The ingroup comprised 59 *Atrax* terminals including two terminals of *Atrax sutherlandi* [19], and 57 terminals of *A. robustus* sensu Gray, 2010 from 45 localities across much of its distribution (Table S1): the Sydney Basin bioregion, the areas around the northern (Newcastle) and southern (Illawarra) distributional limits, and the foothills of the Blue Mountains to the west (Fig. 3). No sequences of *A. yorkmainorum* were available. The outgroup dataset comprised sixteen terminals including one terminal of Atypoidea, which the tree was rooted on; and fifteen terminals of Avicularioidea with taxa chosen according to recent phylogenies for mygalomorph spiders [20–22]: eight terminals of *Hadronyche* (the putative sister-genus of *Atrax*), two terminals of Actinopodidae (the sister-family of Atracidae), one terminal of Domiothelina, and two terminals each of Nemesioidina and Theraphosoidina (Table S1). All sequences were aligned using MAFFT v.7.429 [30, 31] on the CIPRES Science Gateway v.3.3 ([32]: https://www.phylo.org) applying L-INS-i for all loci, and aligned sequences were checked in Mesquite v.3.5 [33]. Aligned molecular markers were concatenated into a single phylogenetic dataset with the dataset partitioned by locus for a total of 75 terminals with 3814 aligned bp. Ingroup sequences were also re-aligned separately to calculate mean interspecific and intraspecific pairwise genetic distances in MEGA11 v.11.0.13 [34].

Phylogenetic analyses were carried out using both Maximum Likelihood (ML) and Bayesian Inference (BI) approaches. A ML analysis was performed in RAxML-HPC2 v.8.2.12 [35, 36] on CIPRES applying either the GTRGAMMA or GTRCAT model to search for the best scoring ML tree with 1000 rapid bootstrap iterations [36]. The BI analysis was performed in MrBayes v.3.2.7a [37] on CIPRES after inferring twenty-four substitution models for each gene in jModelTest2 v.2.1.6 [38, 39] on CIPRES, and choosing the best-fit model based on the Akaike Information Criterion. Four Markov Chain Monte Carlo (MCMC) simulations were run for 25 million generations starting from random trees, sampling every 1000 generations, using a burn-in of 25% for tree-building, and a split frequency < 0.01 to assess chain convergence.

### Divergence time estimation analyses

Divergence times among *Atrax* populations were estimated using BEAST v.1.10.4 [40, 41] on CIPRES with .xml files generated in BEAUTI v.1.10.4 using the compiled phylogenetic dataset described above. The dataset was partitioned by locus except for COI which was partitioned by codon position. Site and clock models were unlinked and the tree model linked across loci. Site models for each locus were chosen using the Bayesian Information Criterion from jModelTest2, and an uncorrelated lognormal molecular clock was applied to all clock models. A birth-death tree prior was applied to the tree model. Since there are no Atracidae fossils, we estimated divergence times via two methods. (i) Considering a dated tree of Mygalomorphae [22], a normal distribution (µ = 155 Ma, σ = 4 Ma) was applied to the split between Nemesioidina and Theraphosoidina. (ii) After testing several arthropod molecular clocks for COI and 16S including Brower [42], Papadopoulou et al. [43], Bidegaray-Batista et al. [44], Abel et al. [45] and Monjaraz-Ruedas et al. [46], the Brower [42] rate for COI (µ = 0.0115, σ = 0.0001), and broad uniform priors for 16S (ucld.min = 0.002, ucld.max = 0.5) and 28S (ucld.min = 0.0001, ucld.max = 0.01), were chosen as the preferred clock rates. Two MCMC chains with different starting seed values were initiated and each chain ran for 500 million generations. Tracer v.1.72 [47] was used to assess convergence of the two chains and to check whether the Effective Sample Size (ESS) for all parameters was above 200. Tree files were combined using Log Combiner after removing 25% of burn-in from each chain, and the maximum clade credibility tree was obtained using Tree Annotator.

### Morphological work

Specimens deposited in the ZMH and the holotype of *A. robustus* were examined using a Leica M125 dissection microscope and images were taken using a custom-made BK Plus System (Dun Inc, Palmyra, PA, U.S.A.) with an integrated Canon camera, a macro lens (65 mm), and Zerene stacking software, v.1.04 (Zerene Systems LLC 2018, Richard, WA, U.S.A.). Digital photos were used to trace proportions and measurements. Female genitalia were excised using a sharp entomological needle, washed in distilled water, digested with Pancreatin solution, cleared in lactic acid and observed under a Leica DM 2500 LED compound microscope, with images taken as above.

The types of *Euctimena tibialis* and *Poikilomorphia montana*, and other historical AMS specimens, were examined under an Olympus XZX-16 microscope, with measurements taken using an eyepiece graticule to 0.1 mm accuracy. Internal genitalia of *P. montana* were digested with Proteinase K and imaged using a Leica system comprising a M205A microscope, DFC 500 camera and LAS 3.8 stacking software (Leica Microsystems, Wetzlar, Germany). Photographs of the dorsal and ventral habitus of *P. montana* were taken with a Canon 600D MkII camera and a Laowa 100 mm macro lens. Images were combined using Helicon Focus (HeliconSoft, Kharkiv, Ukraine) and post-image processing carried out with Topaz AI Sharpen (Topaz Labs, Dallas, TX, U.S.A.).

Scanning electron microscope (SEM) imaging of male genital structures was achieved by dehydration using ethanol solution from 70 to 100% and subsequent transfer to Hexamethyldisilizane (HMDS 99%) for 3 h. The Hitachi Tabletop Microscope TM 4000 Plus (Hitachi, Germany branch) at the ZMH was used for imaging after mounting the structures on a standard SEM stub. All measurements are given in millimetres (mm); total length = carapace length + opisthosoma length.

Locality data for older specimens was georeferenced and such coordinates are indicated in square brackets. To protect species from overharvesting, GPS coordinates are presented only to the nearest minute, or completely obscured for the Newcastle species. Distribution maps were created using QGIS v.3.14.16 (https://www.qgis.org) by superimposing locality records on elevation data ([23]: GMTED2010; https://www.usgs.gov/coastal-changes-and-impacts/gmted2010)*;* lakes and rivers from Geoscience Australia [24]; and borders and coastlines from Natural Earth (https://www.naturalearthdata.com)*.* The new species is registered on ZooBank (http://zoobank.org/)*.* In the following sections, we provide the results of the phylogenetic analyses before we use these data to inform the taxonomic hypotheses and evaluate morphological differentiation within a molecular context.

## Results

### Molecular results

ML and BI phylogenetic trees were largely congruent with minor differences observed in outgroup topology and intraspecific relationships. *Atrax* was recovered as monophyletic with high bootstrap support (100%; Fig. S1). Within *Atrax*, *A. sutherlandi* formed a monophyletic group (96%) sister to *A. robustus* sensu Gray, 2010, which was in turn split into three reciprocally monophyletic groups with mostly high bootstrap/posterior probability values (BT/PP). These three molecular clades correspond to quantifiable levels of morphological divergence and we describe them as distinct species below: *A. robustus* (redefined here; BT/PP: 92%/0.99), *A. montanus* (re-validated here; 45%/0.69), and *A. christenseni* sp. nov. (100%/0.99). *Atrax robustus* was sister to *A. montanus* with high support (81%/0.99), and *A. christenseni* formed a clade sister to (*A. robustus* + *A. montanus*).

Genetic variation within *A. robustus* sensu Gray, 2010 was found to be geographically structured, with specimens from neighbouring localities being more similar genetically. Mean interspecific pairwise genetic distances were recovered as follows: 13.2% (COI) and 7.1% (16S) between *A. montanus* and *A. christenseni*; 13.5% (COI) and 8.5% (16S) between *A. christenseni* and *A. robustus*; and 10.1% (COI) and 4.8% (16S) between *A. robustus* and *A. montanus.* Mean intraspecific genetic distances were recovered as: 1.3% (COI) and 0.8% (16S) for *A. christenseni*; 5.8% (COI) and 2.4% (16S) for *A. montanus*; and 2.6% (COI) and 1.7% (16S) for *A. robustus.* Mean inter- and intraspecific pairwise differences for 28S were < 0.0% across all species.

**Fig. 4:**
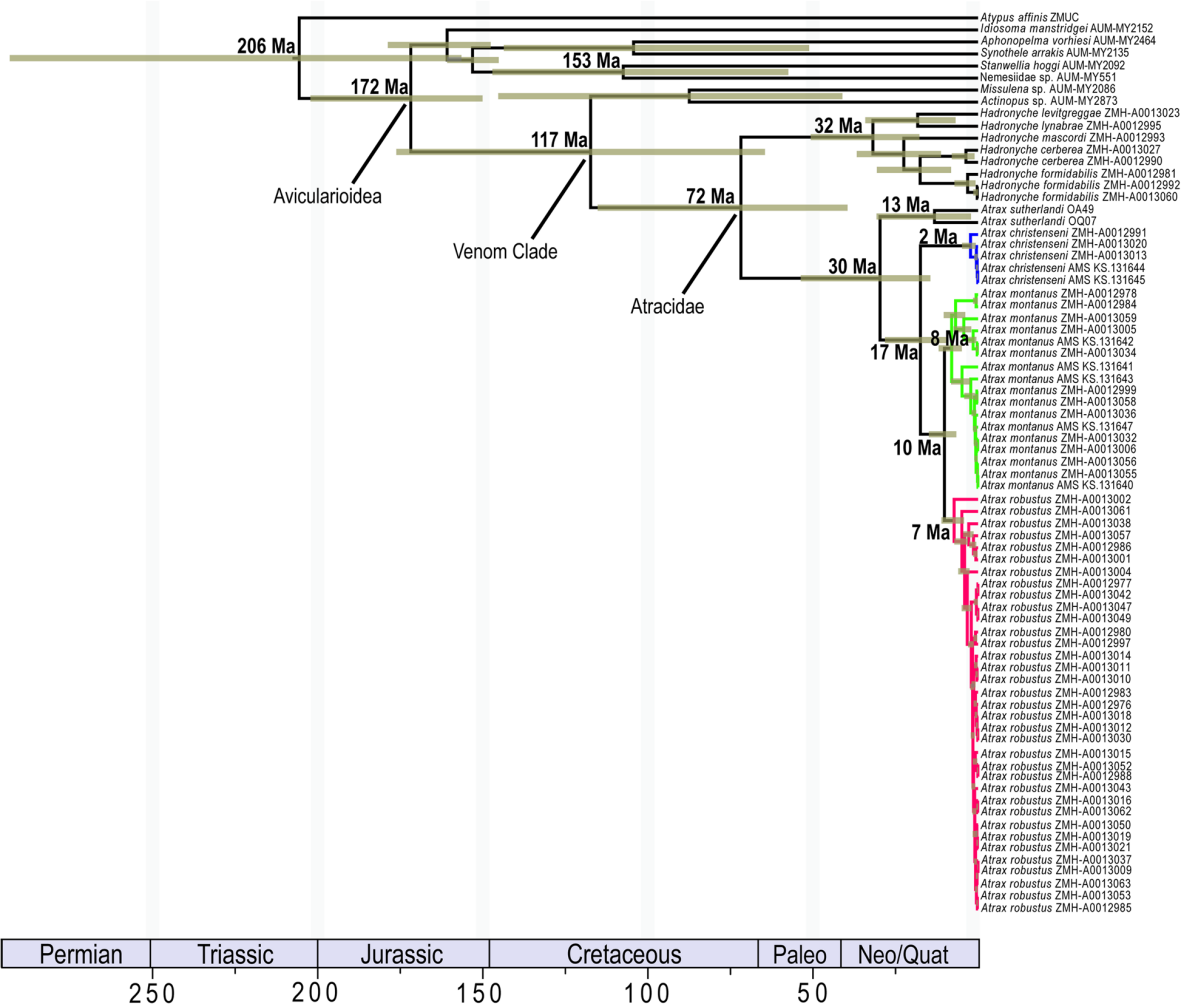
Dated phylogeny of *Atrax* O. Pickard-Cambridge, 1877 based on BEAST analysis of 75 terminals and three genetic markers (COI, 16S and 28S) for a total of 3814 aligned bp. Divergence dates and 95% HPD bars indicated at nodes. Geological time scale includes the following periods: Permian, Triassic, Jurassic, Cretaceous, Paleogene and Neogene/Quaternary (Neo/Quat)

The topology of the divergence time tree was similar to ML and BI trees (Fig. 4). All ESS values were above 100 and only two parameters (28S.ucld.mean/28S.coefficientofvariation) had ESS values below 200. According to the dated tree, *Atrax* diverged from *Hadronyche* in the Late Cretaceous (72 Ma, 95% HPD: 115–40 Ma). Speciation within *Atrax* began in the Oligocene (30 Ma; 95% HPD: 54–14 Ma), whereas diversification of each *Atrax* species began in the late Miocene (13–2 Ma).

### Morphological and combined results

**Fig. 5:**
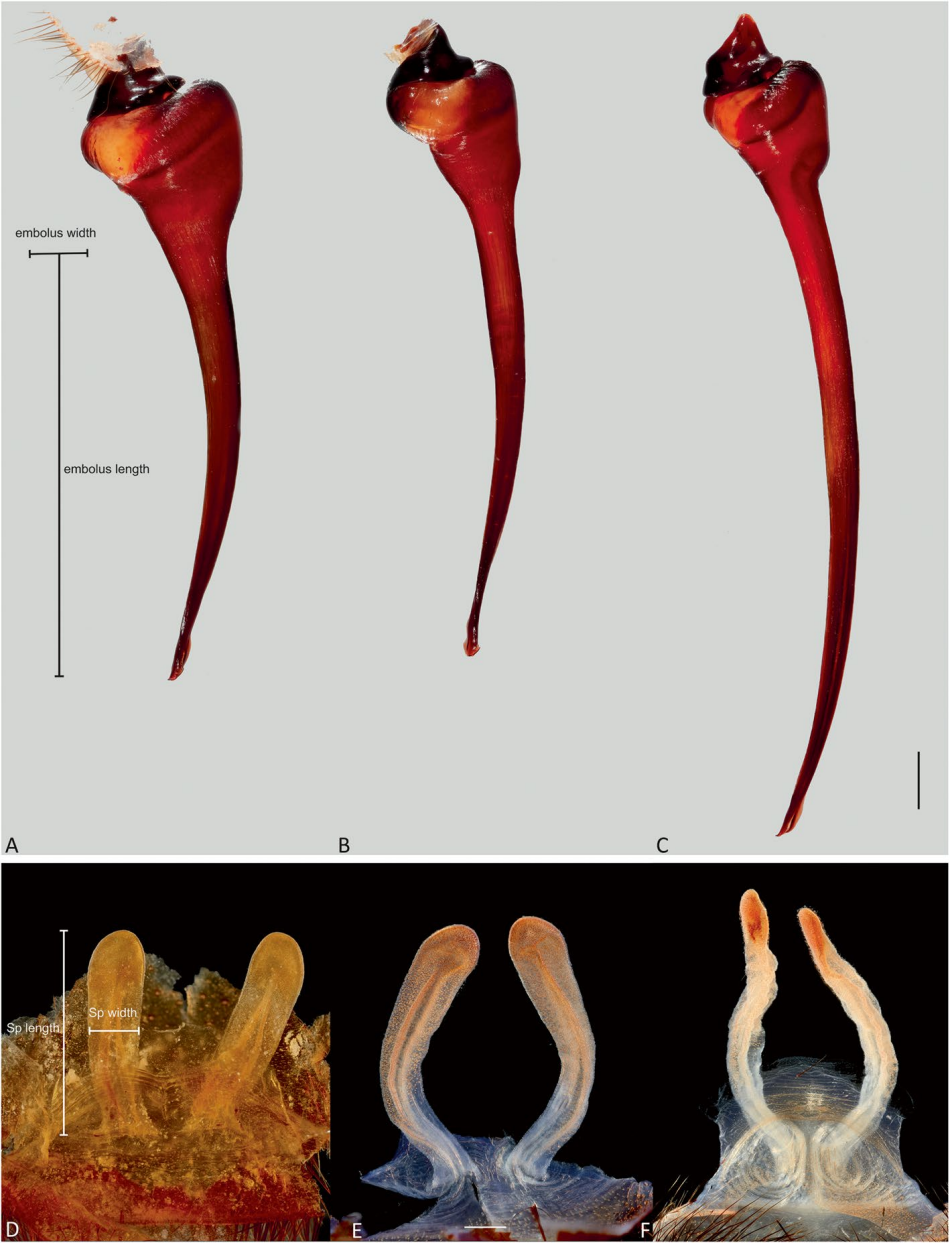
Diagnostic characters of *Atrax* O. Pickard-Cambridge, 1877 species, including male bulb and embolus (**A–C**) in lateral view, and female spermathecae (**D–F**) in dorsal view. **A**,** D. ***Atrax robustus* O. Pickard-Cambridge, 1877: **A.** ♂ (ZMH-A0013030), Mona Vale; **D.** Holotype ♀ (BMNH). **B**,** E. ***Atrax montanus* (Rainbow, 1914): **B.** ♂ (AMS KS.131647), Symbio; **E.** ♀ (ZMH-A0012999), Symbio. **C**,** F. ***Atrax christenseni* sp. nov.: **C.** Holotype ♂ (AMS KS.131644); **F.** Allotype ♀ (AMS KS.131645). Scale bars = 0.5 mm. Annotations indicate measurement points for diagnostic ratios

Genitalic morphology of both males and females supports the molecular findings and fills in the geographical gaps in molecular sampling (Figs. 3 and 5). *Atrax robustus* (64 specimen examinations and 35 terminals/26 localities) is found both north and south of Sydney Harbour across the northern and southern Sydney Basin bioregion. *Atrax montanus* (44 specimen examinations and 17 terminals/14 localities) occurs primarily west and south of *A. robustus*, with records reaching as far south as Stanwell Tops and extending west into the Blue Mountains and some into Central Coast locations. *Atrax robustus* and *A. montanus* overlap in distribution in several areas and we found some specimens (particularly females) from overlap regions to possess genitalia that are somewhat intermediate between *A. robustus* and *A. montanus*, suggesting that hybridization may occur. *Atrax christenseni* included nineteen specimen examinations and five terminals from five localities, and was the most northerly distributed species with all samples collected from a 25 km radius around Newcastle.

### Systematics

**Infraorder** Mygalomorphae Pocock, 1892.

**Family** Atracidae Hogg, 1901.

**Genus ***Atrax* O. Pickard-Cambridge, 1877.

*Atrax robustus* O. Pickard-Cambridge, 1877

Figures 1, 2C and D, 3, 4, 5A and D, 6, 7, 8, 9, 17A, C and E and 18A and B.

**Fig. 6:**
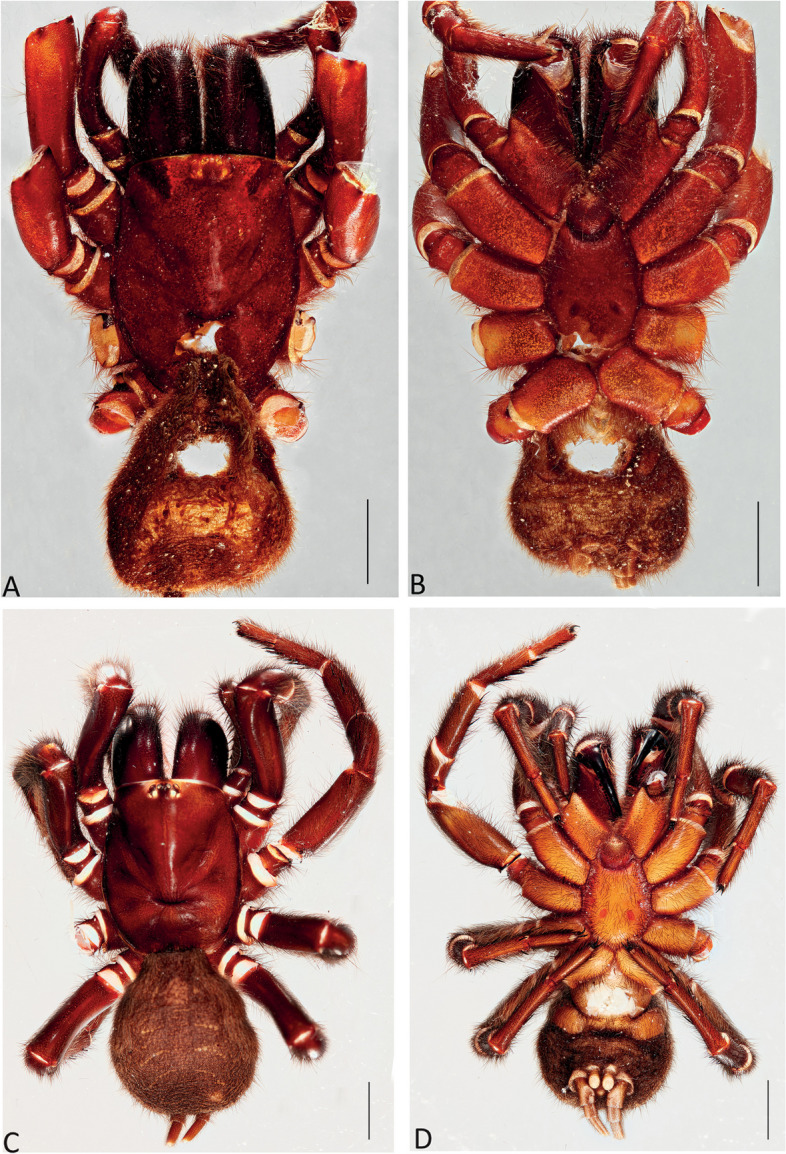
*Atrax robustus* O. Pickard-Cambridge, 1877, dorsal (**A**,** C**) and ventral (**B**,** D**) habitus. **A**,** B.** Holotype ♀ (BMNH). **C**,** D.** ♀ (ZMH-A0013037), Hornsby. Scale bars = 5.0 mm

**Fig. 7:**
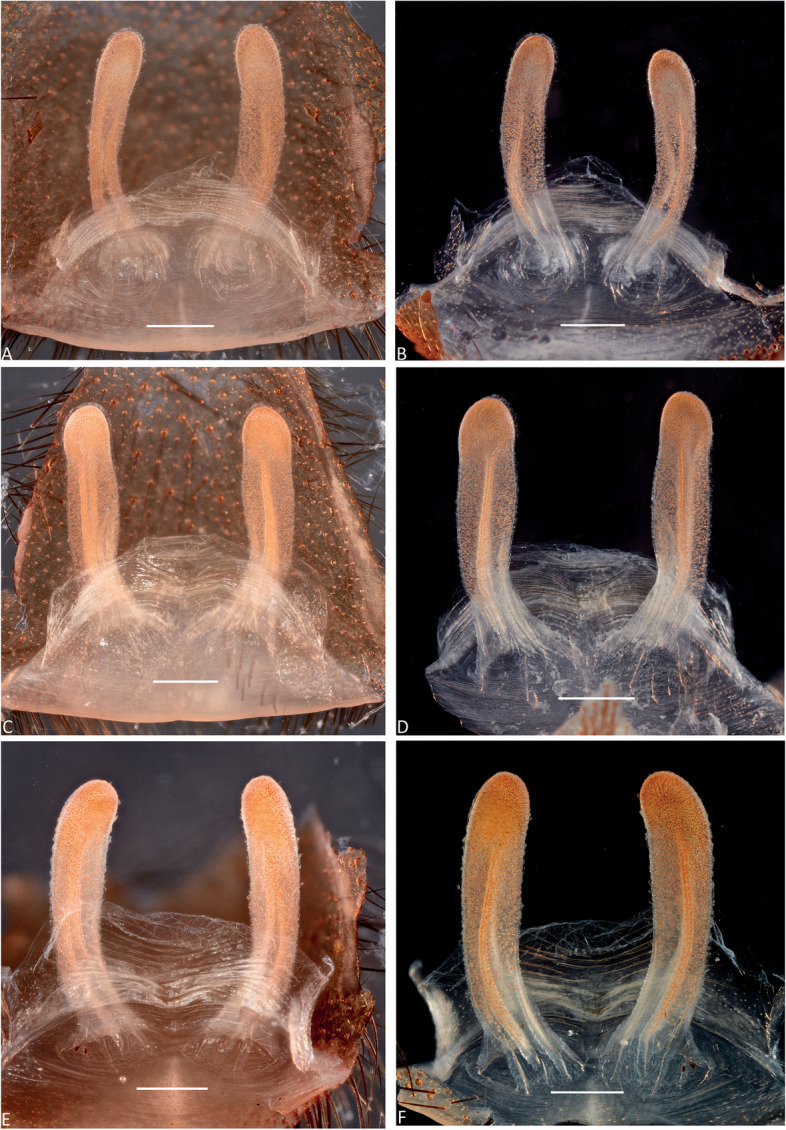
*Atrax robustus* O. Pickard-Cambridge, 1877, female internal genitalia, dorsal (**A**,** C**,** E**), ventral (**B**,** D**,** F**) views. **A**,** B.** ♀ (ZMH-A0013011), Asquith. **C**,** D.** ♀ (ZMH-A0013014), Terrey Hills. **E**,** F.** ♀ (ZMH-A0013037), Hornsby. Scale bars = 0.5 mm

**Fig. 8:**
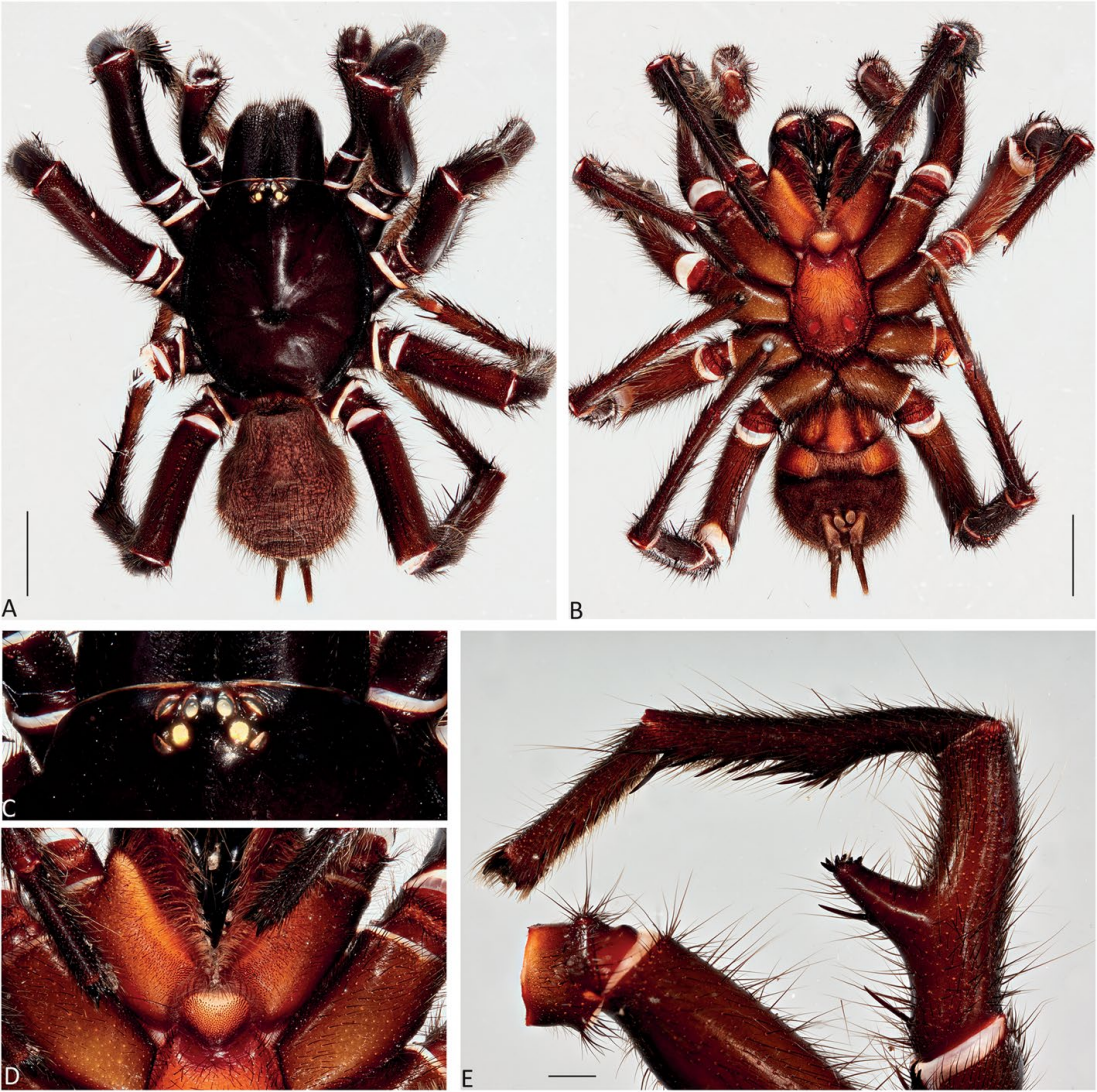
*Atrax robustus* O. Pickard-Cambridge, 1877, Mona Vale, ♂ (ZMH-A0013030), dorsal (**A**) and ventral (**B**) habitus, eyes (**C**), labium and endites (**D**), male leg II (tibia, metatarsus and tarsus), retrolateral view (**E**). Scale bars: **A**,** B** = 5.0 mm; **E** = 1.0 mm

**Fig. 9:**
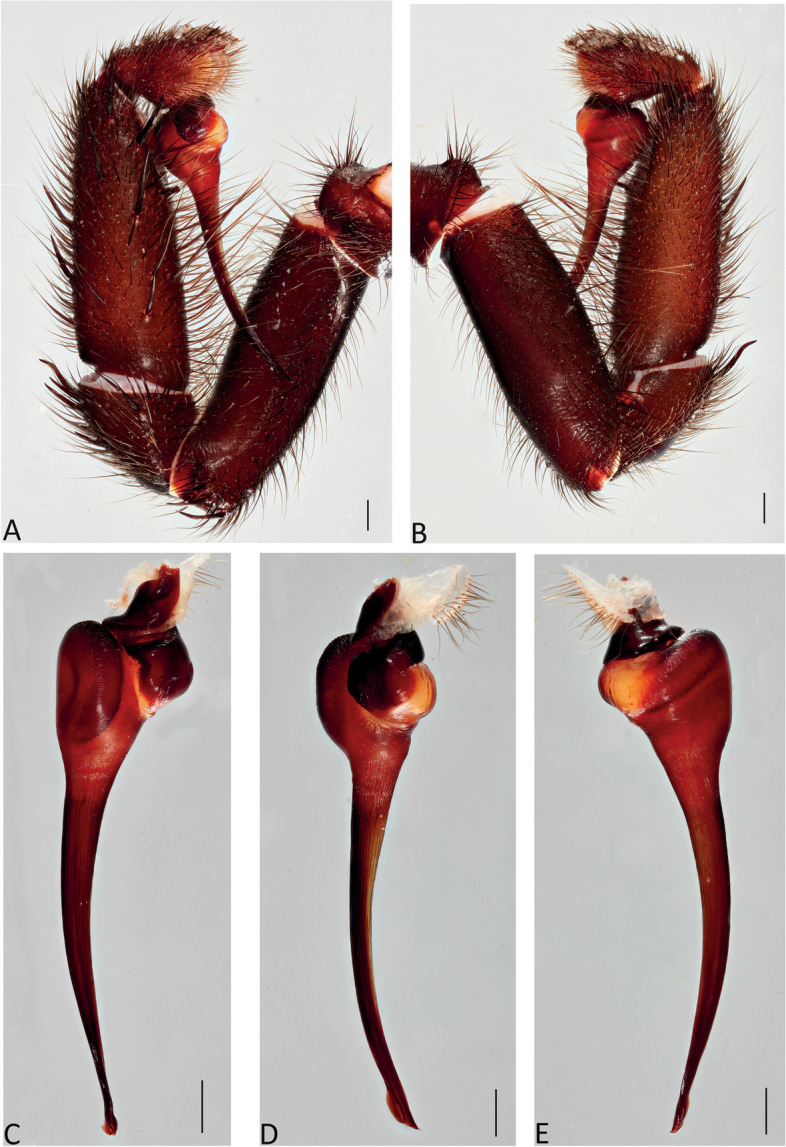
*Atrax robustus* O. Pickard-Cambridge, 1877, Mona Vale, ♂ (ZMH-A0013030). **A**,** B.** Palp, prolateral (**A**) and retrolateral (**B**) views. **C–E.** Bulb and embolus, prolateral (**C**), ventral (**D**) and retrolateral (**E**) views. Scale bars: **A–E** = 0.5 mm

*Atrax robustus* O. Pickard-Cambridge [9]: 27, pl. 6, f. 1 (description female); Hogg [48]: 273, f. 39 (female); Musgrave [11]: 33; Main [14]: 40–42, f. A–F (male); Nishikawa [49]: 179, f. 1–4 (male); Main [15]: 41; Gray [50]: 441, pl. 7; Gray [51]: 314; Gray [16]: 114, 115 (in part); Gray [17]: 299, f. 1A, C, 4A–H, 5A–G, 6A–K (male and female, in part).

*Euctimena tibialis* Rainbow [10]: 249, f. 58–60 (description male) (synonymised by Musgrave [11]: 34, 37, 38).

**Type Material. AUSTRALIA**: *Atrax robustus*: Holotype ♀ (BMNH) (formerly dried, now in 75% ethanol), 71 Neu Holland [Australia] [examined]. *New South Wales*: *Euctimena tibialis*: Holotype ♂ (AMS KS.4092), Turramurra [33º44’S 151º08’E], obtained from under a log [examined]. 

**Material Examined. AUSTRALIA**: *New South Wales*: Ashfield [33º53’S 151º07’E], Sydney, 20 Dec. 1930, E. Hudson, 1♀ (AMS KS.4930); Asquith [33º41’S 151º06’E], 2 Nov. 2018, K. Christensen, 1♀ (ZMH-A0013011); Avoca Beach [33º28’S 151º26’E], 20 Sept. 1968, N. Tweedale, 1♀ (AMS KS.5017); Balmain [33°52’S 151°11’E], Sydney, Jan. 1996, 1♂ (AMS KS.45611); Bankstown [33°55’S 151°02’E], P. Finn, 1♀ (AMS KS.4943), 14 Apr. 1965, 1♂ (AMS KS.4902); Baulkham Hills [33º46’S 150º59’E], 19 Jan. 1973, M. Gray, 1♀ (AMS KS.5139), 17 Mar. 2020, K. Christensen, 1♀ (ZMH-A0013024); Beecroft [33º46’S 151º03’E], dug out of the ground, 25 Apr. 2020, B. Jones, 1♀ (ZMH-A0013019), 26 Apr. 2020, B. Jones, 1♂ (ZMH-A0013021); Berowra Valley National Park, Benowie Walking Track, 33º40’S 151º05’E, hand collected, 71 m, 17 Mar. 2020, D. Harms & H. Smith, 1juv. (ZMH-A0013062); Bidjigal [Biddigal] Reserve, 33º45’S 151º01’E, 11 Jan. 2020, B. Jones, 1juv. (ZMH-A013043); Bilgoa Plateau [33º38’S 151º19’E], 27 Jan. 2020, K. Christensen, 1♂ (ZMH-A0013012); Bilpin [33º30’S 150º31’E], K. Christensen, 1juv. (ZMH-A0013061), 17 Jan. 1930, E.M. Hunt, 1♂ (AMS KS.1148); Brookvale [33º46’S 151º16’E], K. Christensen, 1♀ (ZMH-A0013015); Central Coast, 1♀ (AMS KS.130960); Central Coast area, 24 Oct. 2019, K. Christensen, 1♂ (ZMH-A0012986); Clifton Gardens [33°50’S 151°15’E], Sydney, 20 Jun. 1927, C.A. Monticone, 1♂ (AMS KS.4378); Cook St. Trail, 33º46’S 151º13’E, 15 Jan. 2020, B. Jones, 1♀ (ZMH-A0012988), 1juv. (ZMH-A0013052); Cumberland State Forest, 33º45’S 151º02’E, 10 Jan. 2020, B. Jones, 1juv. (ZMH-A0013053); Deep Creek Reserve, 33º43’S 151º16’E, 15 Jan. 2020, B. Jones, 1♀ (ZMH-A0012977), 1juv. (ZMH-A0013042); Dural [33°41’S 151°02’E], Mar. 1981, 1♂ (AMS KS.7470); Elizabeth Bay [33º52’S 151º14’E], Sydney, 9 Jan. 1958, F. Siider, 1♀ (AMS KS.4989), 5 May 1981, D.T. Cave, 1♂ (AMS KS.7477); Galston [33º39’S 151º03’E], 16 Feb. 1986, G. Jackson, 1♀ (AMS KS.16443), 19 Apr. 2019, K. Christensen, 1♀ (ZMH-A0013016); Galston Gorge [33°40’S 151°05’E], 25 May 2003, 1♂ (AMS KS.88193); Garigal National Park, 33º43’S 151º11’E, 15 Jan. 2020, B. Jones, 2juvs. (ZMH-A0013047, ZMH-A0013049); Gordon [33º45’S 151º09’E], 29 Apr. 1973, J. Buist, 1♀ (AMS KS.5142); Glenning Valley [32°21’S 151°25’E], 1♂ (AMS KS.128227); Gosford [33º26’S 151º21’E], 1♂ (AMS KS.52486), 9 Jun. 2019, K. Christensen, 1♂ (ZMH-A0013001), 1981, 1♂ (AMS KS.8543); Grays Point [34°04’S 151°05’E], Jun. 1992, K. Swarbrick, 1♂ (AMS KS.31961); Hornsby [33º42’S 151º06’E], 31 Mar. 1941, 1♀ (AMS KS.4363), 2 Feb. 1996, 1♀ (AMS KS.48998), 3 Oct. 2019, K. Christensen, 1♀ (ZMH-A0013037), Hornsby Hospital [33º42’S 151º07’E], 7 Aug. 2018, K. Christensen, 1♀ (ZMH-A0013004), 4 Sept. 2018, K. Christensen, 1♀ (ZMH-A0013010), 28 Jun. 2019, K. Christensen, 1♀ (ZMH-A0013009); via Hornsby Hospital [33º42’S 151º07’E], 10 Dec. 2019, K. Christensen, 1♀ (ZMH-A0012985); Hornsby Heights [33°40’S 151°06’E], 5 Mar. 1977, A. Young, 1♂ (AMS KS.661); Hurstville [33°58’S 151°06’E], 5 Jun. 1972, A. Osten, 1♂ (AMS KS.4949), 2 Dec. 1993, N. Parsons, 1♀ (AMS KS.40633); Hurstville Sth [33°58’S 151°06’E], 8 Nov. 1971, G. Dwyet, 1♂ (AMS KS.4928); Kilcare Beach [Kilcare, 33º32’S 151º22’E], Gosford area, 4 Oct. 1977, F. Bradfield, 1♀ (AMS KS.3610); Kincumber [33°28’S 151°24’E], 16 Feb. 1978, B. Valentine, 1♂ (AMS KS.50289); Ku-ring-gai Chase National Park, 33º40’S 151º13’E, 15 Jan. 2020, B. Jones, 1♀ (ZMH-A0012997), 1juv. (ZMH-A0012980); Kurrajong Heights [33º31’S 150º38’E], 24 Feb. 1980, 1♀ (AMS KS.4622); Lakemba [33°55’S 151°04’E], 7 Jan. 1948, 1♂ (AMS KS.4944); Lane Cove [33º49’S 151º10’E], 14 Jul. 1930, Lee, 1♀ (AMS KS.4407), 5 Feb. 1934, R. Cooney, 1♀ (AMS KS.4398), 24 May 1973, L. Mackiewicz, 1♀ (AMS KS.5143), 21 May 1993, Ryden, 1♂ (AMS KS.35086); Lane Cove National Park, 33º46’S 151º06’E, 10 Jan. 2020, B. Jones, 1juv. (ZMH-A0013050); Lindfield [33º47’S 151º10’E], 29 Sept.1962, M. Gregg & V. Gregg, 1♀ (AMS KS.5031); Manly [33º48’S 151º17’E], 7 Apr. 1930, T. Iredale, 1♀ (AMS KS.4866); Matcham [33º25’S 151º26’E], brick pile, 30 Jun. 2020, K. Christensen, 1♂ (ZMH-A0013002), 18 Oct. 2020, K. Christensen, 1♂ (AMS KS.130966); Matraville South [33º58’S 151º14’E], 20 Oct. 1952, A. Brown, 1♀ (AMS KS.4907); Meadowbank [33º49’S 151º05’E], Mar. 1963, R.H. Hall, 1♀ (AMS KS.4238); Mona Vale Vet [Mona Vale Vet Hospital, 33º41’S 151º18’E], 11 Mar. 2020, K. Christensen, 1♂ (ZMH-A0013030); Mosman [33º50’S 151º15’E], North Sydney, 1♂ (AMS KS.4193 – labeled “compared with type” of *Euctimena tibialis*); Mosman [33º50’S 151º15’E], 1 Dec. 1930, A. Gould, 1♀ (AMS KS.4237); Mt. Colah [33°40’S 151°07’E], 10 Jun. 1996, 1♂ (AMS KS.45840); Neutral Bay [33°50’S 151°13’E], 1♂ (AMS KS.4009); Parramatta [33°49’S 151°00’E], 25 Mar. 1959, W.G. Ashford, 1♂ (AMS KS.4950); Pymble [33°45’S 151°08’E], 29 Jan. 1993, 1♂ (AMS KS.34398); Roseville [33º47’S 151º10’E], 17 Sept. 1952, A. Simpson, 1♀ (AMS KS.4861); Scotland Island [33º38’S 151º17’E], K. Christensen, 1♀ (ZMH-A0012983), 29 May 1970, C. Mclean, 1♀ (AMS KS.4379), 23 Nov. 2018, K. Christensen 1♀ (ZMH-A0012976); Stanmore [33°54’S 151°10’E], 1949, Jany, 1♂ (AMS KS.4922); Sydney University, Macleay Bldg [33º53’S 151º11’E], 7 Feb. 1985, R. Bradley, 1♀ (AMS KS.50024); Tascott [33°27’S 151°19’E], 1 Nov. 2020, K. Christensen, 1♂ (AMS KS.130962); Telopea [33°48’S 151°03’E], 25 Apr. 1971, K.P. Reid, 1♂ (AMS KS.4971); Terry Hills [Terrey Hills, 33º41’S 151º13’E], 20 Sept. 2019, K. Christensen, 1♀ (ZMH-A0013014); Terrey Hills [33º41’S 151º13’E], 15 Jul. 2020, K. Christensen, 1♀ (ZMH-A0013018); Terrigal [33º27’S 151º27’E], 27 Sept. 2019, K. Christensen, 1♀ (ZMH-A0013038); Wahroonga [33°43’S 151°07’E], 16 Mar. 1930, 1♂ (AMS KS.4028); Warrawee [33º44’S 151º07’E], 9 Apr. 1961, M. Gregg, 1♀ (AMS KS.5032); Waverley [33°54’S 151°15’E], 15 May 1973, Gibson, 1♂ (AMS KS.5146), 3 Jul. 1973, R. Stewart, 1♂ (AMS KS.4972); West Pennant Hills [33º45’S 151º02’E], 15 May 1973, S. Zucker, 1♀ (AMS KS.5130); West Pennant Hills, 33º45’S 151º03’E, hand collected, 111 m, 19 Mar. 2020, D. Harms & B. Buzatto, 1juv. (ZMH-A0013063); Winston Hills [33º47’S 150º59’E], 6 Sept. 1972, J. Deviana, 1♀ (AMS KS.5148), 11 Apr. 1973, J. Deviana & V. Gregg, 1♀ (AMS KS.5133), 4 Apr. 1974, J. Deviana & V. Gregg, 1♂ (AMS KS.5152); Wollongong [34º26’S 150º53’E], Jul. 1974, B. Purdy, 1♀ (AMS KS.5012); Wollstonecraft [33°50’S 151°12’E], 27 Jan. 1993, 1♂ (AMS KS.34392); Wyoming Vet [Wyoming, 33º24’S 151º22’E], 4 Apr. 2020, K. Christensen, 1♂ (ZMH-A0013057); Yagoona [33º54’S 151º01’E], Feb. 1981, 1♀ (AMS KS.8295).

**Fig. 10:**
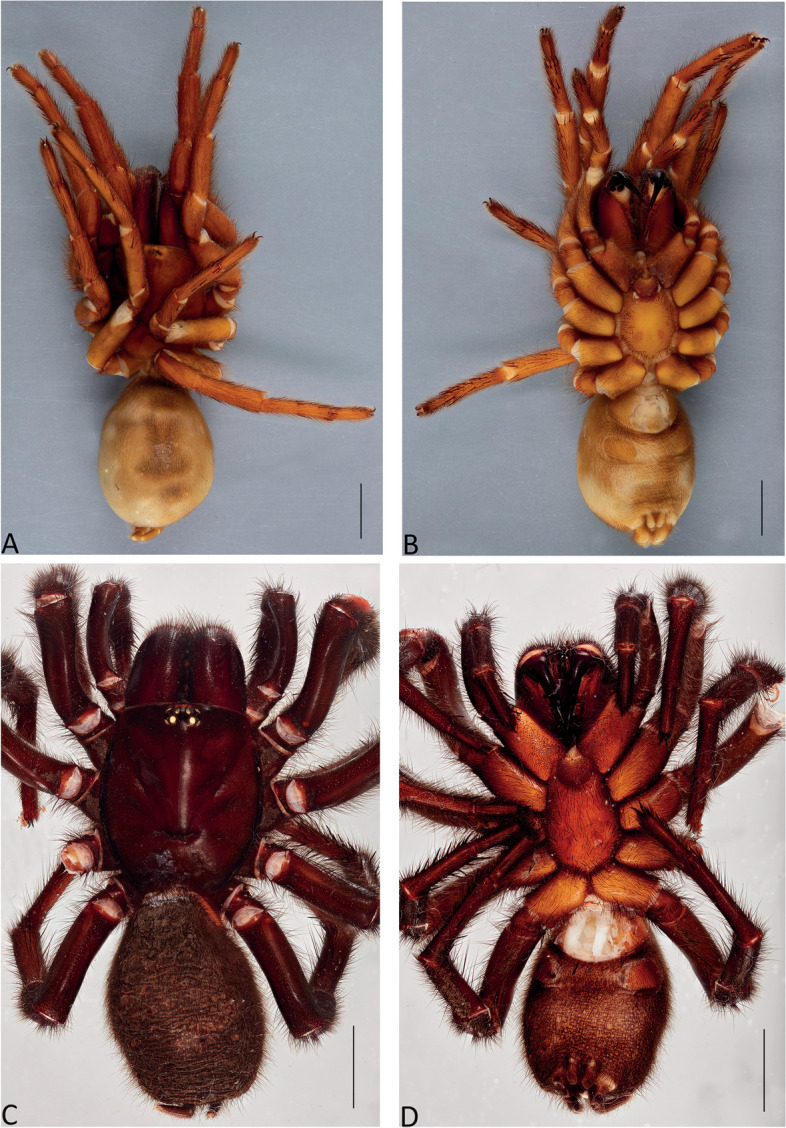
*Atrax montanus* (Rainbow, 1914), dorsal (**A**, ** C**) and ventral (**B**, ** D**) habitus. **A**, ** B.** Holotype ♀ (AMS KS.1039). **C**,** D.** ♀ (AMS KS.131640), Stanwell Tops. Scale bars = 5.0 mm

**Diagnosis.** Adult males of *A. robustus* differ from all congeners by the following combination of characters: carapace length x̅ 10.2 (8.9–13.4); palpal tibia short, 2x longer than wide (Fig. 9); embolus short (6.5x longer than wide) and with smooth curvature (Figs. 5A and 17A, C and E). Males can be further distinguished from *A. montanus* by their shorter embolus (6.5x longer than wide) and wider embolus tip opening (Figs. 5A and 17A, C and E), while in the latter, the embolus is longer (8x longer than wide) and the embolus tip opening is narrower (Figs. 5B and 17B, D and F); from *A. christenseni* sp. nov. by the shorter and broader embolus (6.5x longer than wide), while 12x longer than wide in the latter (Fig. 5C); from *A. sutherlandi* by their embolus longer than the palpal tibia and smoothly curved vs. embolus shorter than palpal tibia and strongly curved (see Gray [17]: fig. 12G); and from *A. yorkmainorum* by the slightly twisted embolus tip that is strongly twisted in the latter ([17]: fig. 10A). Adult females of *A. robustus* differ from all other species by the following combination of characters: carapace length x̅ 11.9 (9.0–14.5); spermathecae straight, 4–5x longer than wide. In *A. sutherlandi* the spermathecae are 3x longer than wide (see Gray [17]: fig. 13F). *Atrax robustus* and *A. montanus* are generally smaller species than *A. christenseni* sp. nov.

**Description. Male (ZMH-A0013030).** Total length: 25.14; carapace length: 13.35; carapace width: 9.17; abdomen length: 11.80 (Fig. 8). **Colouration** (in ethanol): Carapace, endites and sternum dark brown; chelicerae dark brown; abdomen dark grey; legs dark brown. **Carapace**: Broadly oval, pars cephalica slightly elevated; fovea transverse, procurved. **Endites**: With **~** 289 cuspules. **Labium**: With **~** 379 cuspules. **Eyes**: Eye formula 4:4; ocular quadrangle rectangular; AME separated by 1.3x their own diameter; PME separated by 2.1x own diameter. **Sternum**: Longer than wide; three pairs of ovoid sigilla, posterior pair the largest. **Chelicerae**: Rastellum absent; promargin with 15 teeth, retromargin with 16 teeth, furrow with 32 denticles. **Leg measurements**: I 31.01 (8.52/3.79/7.18/7.03/4.49); II 27.75 (7.42/3.56/6.93/6.17/3.67); III 22.89 (6.44/3.02/4.91/5.00/3.52); IV 30.21 (7.89/3.78/6.99/7.10/4.55). **Abdomen**: Ovoid; with four spinnerets. **Genitalia**: Palpal tibia 4.48/2.06; bulb 5.45/1.38. Palpal tibia with four macrosetae prolateral; 2x longer than wide; embolus 6.5x longer than wide; smooth curvature; embolus tip with small flap and pointed tip (Figs. 9, 17A, C and E and 18A and B).

**Female Holotype (BMNH).** Total length: 27.87; carapace length: 13.98; carapace width: 10.29; abdomen length: 13.89 (Fig. 6). **Colouration** (in ethanol specimen desiccated): Carapace and legs reddish; chelicerae reddish-brown; abdomen brown; sternum reddish-brown; labium and endites reddish-brown with black cuspules. **Carapace**: Broadly oval; pars cephalica slightly elevated; fovea transverse, slightly procurved. **Endites**: ~274 cuspules. **Labium**: ~351 cuspules. **Sternum**: Longer than wide; three pairs of ovoid sigilla; posterior pair of sigilla largest, anterior pairs much smaller, subequal. **Chelicerae**: Rastellum absent; promargin with 14 teeth, retromargin with 17 teeth, furrow with ~ 16 denticles. **Eyes**: Eye formula 4:4; ocular quadrangle rectangular; AME separated by 1x their own diameter; PME separated 3.2x by their own diameter. **Leg measurements**: Too damaged. **Abdomen**: Ovoid; with four spinnerets. **Genitalia**: Internal genitalia with two long spermathecae (4x longer than wide), straight (Fig. 7).

**Female (ZMH-A0013037).** Total length: 27.50; carapace length: 11.55: carapace width: 9.67; abdomen length: 15.95 (Fig. 6). **Colouration** (in ethanol): Carapace, endites and sternum dark reddish-brown; chelicerae dark brown; abdomen dark grey; legs dark reddish-brown. **Carapace**: Broadly oval, pars cephalica slightly elevated; fovea transverse, procurved. **Endites**: With **~** 290 cuspules. **Labium**: With **~** 349 cuspules. **Eyes**: Eye formula 4:4; ocular quadrangle rectangular; AME separated by 1.1x their own diameter; PME separated by 3x own diameter. **Sternum**: Longer than wide; three pairs of ovoid sigilla, posterior pair the largest. **Chelicerae**: Rastellum absent; promargin with 14 teeth, retromargin with 15 teeth, furrow with 22 denticles. **Leg measurements**: I 28.45 (8.22/3.79/6.18/6.03/4.23); II 27.28 (7.44/3.58/6.17/6.07/4.02); III 22.77 (6.32/3.02/4.90/4.56/3.97); IV 27.19 (7.85/3.15/5.98/6.00/4.21). **Abdomen**: Ovoid; with four spinnerets. **Genitalia**: Internal genitalia with two long spermathecae (4–5x longer than wide); spermathecae straight; circular openings almost touching (Fig. 7).

**Variation.** Male carapace length: 8.9–13.4, x̅ 10.2, *n* = 34; total length: 25.14–29.26, x̅ 27.20, *n* = 5; female carapace length: 9.0–14.5, x̅ 11.9, *n* = 21; total length: 27.50–39.32, x̅ 33.41, *n* = 5. Female spermathecae vary in length, width and degree of curvature; some specimens have thin and slightly divergent spermathecae whilst others are parallel, some diverge slightly at the base before straightening distally.

**Distribution.** Based on material examined in the present study, *A. robustus* was historically found in the northern (Central Coast) and southern Sydney Basin bioregion between Georges River (south) and Tuggerah Lake (north), extending as far west as Baulkham Hills near the southern end of its distribution (Fig. 3). Some records are also known from the Blue Mountains and Wollongong. More recent records from iNaturalist (https://www.inaturalist.org/) and from the AMS collection indicate that the range of *A. robustus* in the area south of the Parramatta River has been reduced and is limited to the Bankstown–Mortdale area.

**Natural History.** This species is associated with open and closed sclerophyll forests and woodlands, often near gullies with rainforest elements. It seems quite adaptable and is also found in gardens, parks or industrial areas, as long as the area is partially shaded (Fig. 2C, D), has some degree of permanent moisture, and can provide a refuge from fire and excessive heat. Main [14] and Bradley [52] provide data on seasonal activity, Levitt [53] biological data on specimens in captivity, Duran et al. [12] on defensive behaviour, and Frank et al. [13] a description of mating behaviour in captivity. Note that older publications might also allude to *A. montanus*, specifically if they refer to specimens from “damp closed forests” [52].

**Taxonomic Remarks.** The taxonomic history of *Atrax robustus* is complex and needs some explanation. The original description by O. Pickard-Cambridge [9] is brief and was based on a single, poorly preserved, dried female specimen. Hogg [48] re-described this species based on several female specimens from Queensland and New South Wales in the BMNH collections using colour patterns and eye ratios, with his records from Queensland almost certainly representing misidentified *Hadronyche* females. Rainbow [10] described males of *A. robustus* from Turramurra and Mosman (Sydney) as a distinct genus and species, *Euctimena tibialis*. In his work on venomous Australian spiders, Musgrave [11] examined a large black spider that caused the death of a young child in Thornleigh and matched it not only with Rainbow’s holotype of *E. tibialis*, but also used somatic morphological characters and collection data to match this male with females of *Atrax robustus*. The taxonomy was further confused by weak generic concepts meaning that Koch [54] established *Hadronyche* as a second genus for Australian funnel-web spiders but both *Atrax* and *Hadronyche* were diagnosed so poorly that many more species were described in these genera over the years, or shifted between them. Main [14] recognized six species of *Atrax* and Gray [50] added three additional species before he transferred all species except *A. robustus* to *Hadronyche* [17]. Main [15] transferred the Blue Mountains species *Poikilomorphia montana* Rainbow, 1914 to *Atrax* (as *A*. *montanus*), but this species was later synonymised with *A. robustus* by Gray [16] and is re-validated here. In his major taxonomic review of funnel-web spiders, Gray [17] not only re-defined and re-diagnosed *Atrax* and *Hadronyche* and described *Illawarra* as a third genus, but also described and diagnosed three species of *Atrax*, with *A. robustus* differing from *A. sutherlandi* by a more elongate embolus and from *A. yorkmainorum* by a larger body size and a relatively spinose pedipalpal tibia. In his re-description of *A. robustus*, Gray [17] notes that specimens from the Newcastle region are larger than other individuals and have a longer embolus. These specimens are described here as *A. christenseni* sp. nov. Overall, Gray [17] had an extensive collection of specimens at his disposal but was taxonomically cautious in the absence of clear-cut morphological features and the continuum of morphological characters. He was never able to examine the holotype of *A. robustus* (BMNH), which had unclear locality data and we have amended his original description with images of the holotype.

*Atrax montanus* (Rainbow, 1914), status revised

Figures 2A, 3, 4, 5B and E, 10, 11, 12, 13, 17B, D and F and 18C and D.

**Fig. 11:**
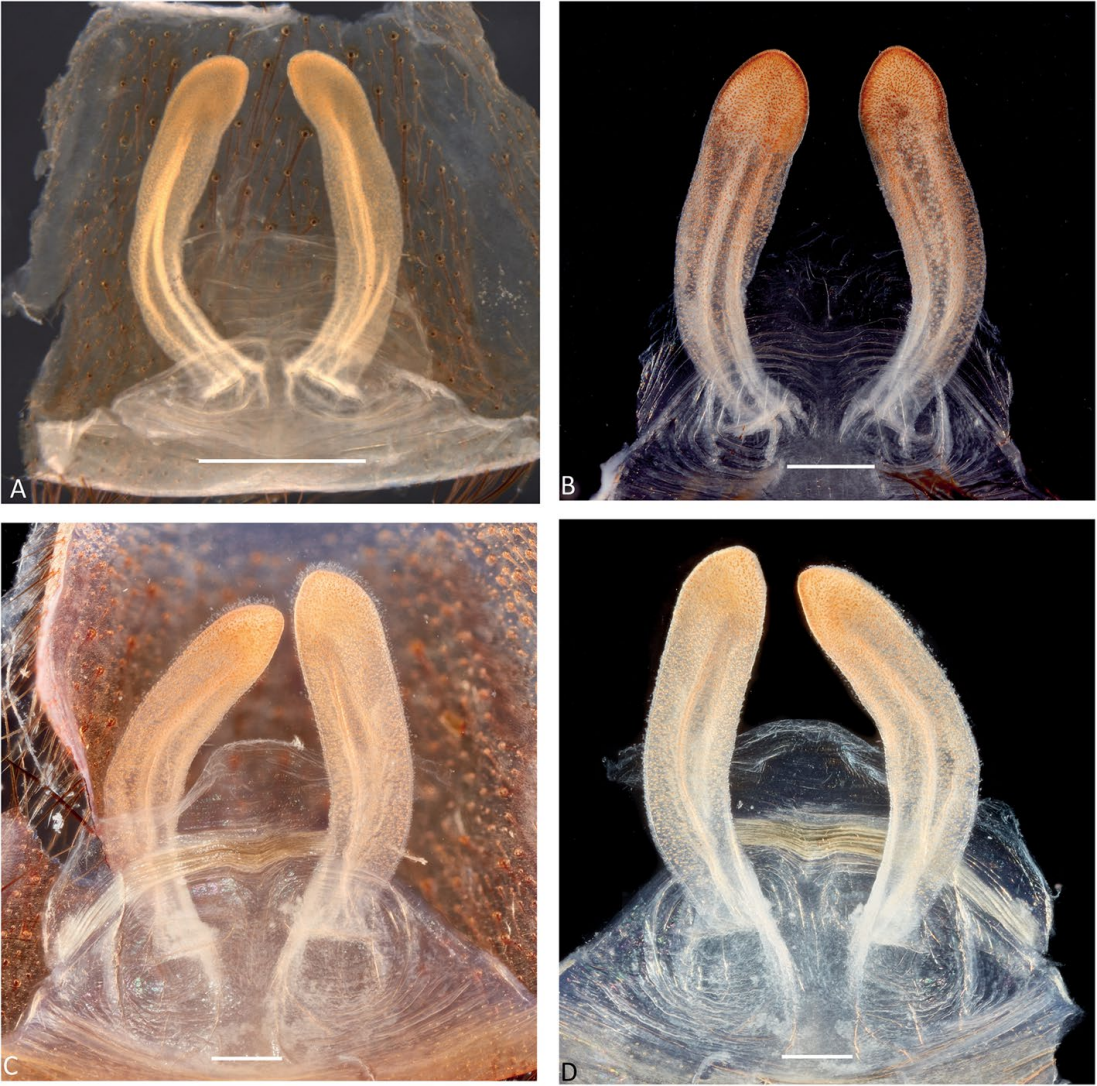
*Atrax montanus* (Rainbow, 1914), dorsal (**A**,** C**) and ventral (**B**,** D**) female internal genitalia. **A.** Holotype ♀ (AMS KS.1039). **B.** ♀ (AMS KS.131640), Stanwell Tops. **C**,** D.** ♀ (AMS KS.131642), Springwood. Scale bars: **A** = 1.0 mm; **B–D** = 0.5 mm

**Fig. 12:**
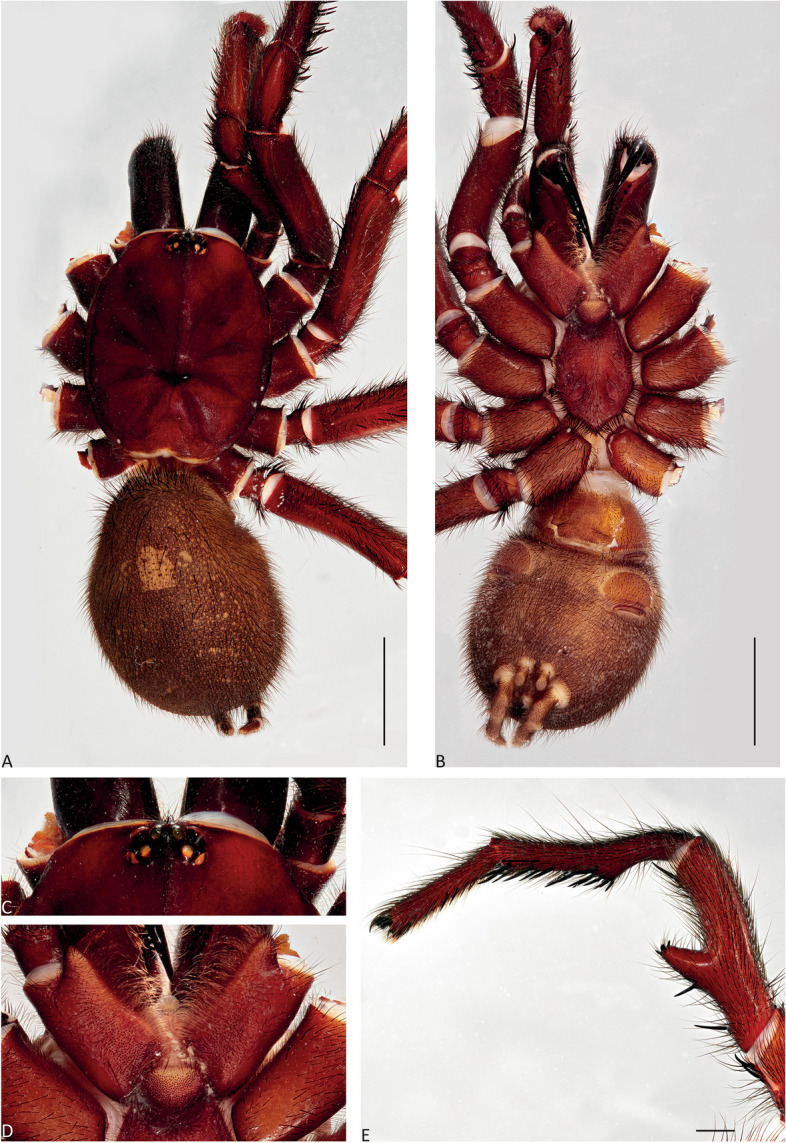
*Atrax montanus* (Rainbow, 1914), Symbio, ♂ (AMS KS.131647), dorsal (**A**) and ventral (**B**) habitus, eyes (**C**), labium and endites (**D**), male leg II (tibia, metatarsus and tarsus), retrolateral view (**E**). Scale bars: **A**,** B** = 5.0 mm; **E** = 1.0 mm

**Fig. 13:**
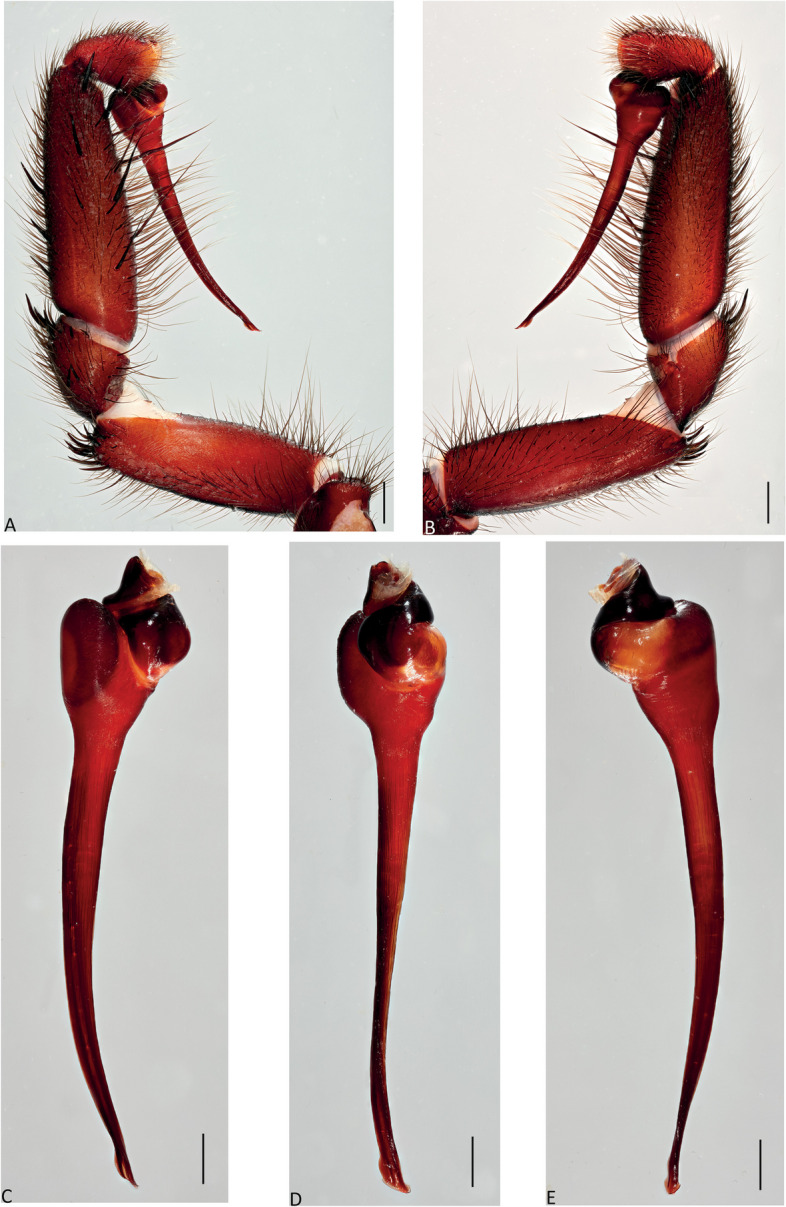
*Atrax montanus* (Rainbow, 1914), Symbio, ♂ (AMS KS.131647). **A**,** B.** Palp, prolateral (**A**) and retrolateral (**B**) views. **C–E.** Bulb and embolus, prolateral (**C**), ventral (**D**) and retrolateral (**E**) views. Scale bars: **A**,** B** = 1.0 mm; **C–E** = 0.5 mm

*Poikilomorphia montana* Rainbow [10]: 265, f. 71, 72 (description female).

*Atrax montanus*: Main [15]: 41.

*Atrax robustus*: Gray [16]: 114, 115 (in part; *P. montana* synonymised with *A. robustus*); Gray [17]: 299, f. 1A, C, 4A–H, 5A–G, 6A–K (in part).

**Type material. AUSTRALIA: **Holotype ♀ (AMS KS.1039), Blue Mountains, Wentworth Falls [33º43’S 150º22’E], Jamieson Valley [examined]. 

**Material Examined. AUSTRALIA**: *New South Wales*: Barr Point [33°31’S 151°10’E], Hawkesbury River, 31 Mar. 1986, Callaghan, 1♂ (AMS KS.16538); Belimba Park [34º04’S 150º33’E], 10 Aug. 2020, K. Christensen, 1♀ (ZMH-A0013058); Bilpin [33º30’S 150º31’E], Sept. 1929, E.M. Huny, 1♀ (AMS KS.1221); Blackheath [33º38’S 150º17’E], 24 Apr. 1948, R. Mckay, 1♀ (AMS KS.1674); Bowral Old South Rd. [ca. 34º29’S 150º26’E], 13 Jun. 1973, M.C. Kinlay, 1♀ (AMS KS.4887); Burraneer Bay [34°04’S 151°08’E], 15 Dec. 1972, 1♂ (AMS KS.4961); Camden [34°03’S 150°42’E], 11 Jun. 1950, V.C. Levitt, 1♀ (AMS KS.5000), 21 Feb. 1974, G. Seymour, 1♂ (AMS KS.1154); Castle Hill [33º39’S 151º03’E], Galston, 26 Mar. 1973, J. Mutton, 1♀ (AMS KS.5128); Clifton Gardens [33º51’S 151º15’E], Sydney, 21 Apr. 1961, Bradley, 1♀ (AMS KS.4030); Colo Vale [34º24’S 150º29’E], 1♀ (AMS KS.5001); Cooranbong [33º04’S 151º27’E], Jul. 1933, E.G. King, 1♀ (AMS KS.1124); Coxs River nr. junction of Breakfast Ck., Blue Mountains, 12 Jan. 1997, 1♀ (AMS KS.49700); Cronulla [34º03’S 151º09’E], 20 Jun. 1971, 1♀ (AMS KS.4988), Aug. 1983, 1♀ (AMS KS.13587); Engadine [34º04’S 151º02’E], 26 Feb. 1973, R. Witchard, 1♂ (AMS KS.4917), 18 Jan. 1990, B. Borham, 1♂ (AMS KS.32391), 10 Aug. 2020, K. Christensen, 1juv. (ZMH-A0013036); Faulconbridge [33°42’S 150°32’E], 28 Jan. 1974, J. Crick, 1♂ (AMS KS.3608); Freemans Reach [ca. 33º36’S 150º49’E], near Windsor, Hawkesbury River, 26 Jan. 1982, M. Gray, 1♀ (AMS KS.8868); Glenning Valley [33º21’S 151º25’E], 23 Dec. 2019, K. Christensen, 1♂ (ZMH-A0013059); Guildford West [33º51’S 150º58’E], 27 Aug. 1957, S.R. Kirkwood, 1♀ (AMS KS.4966); Hartley Vale [33°32’S 150°14’E], 8 Dec. 1952, E.S. Miller, 1♂ (AMS KS.1160); Hazelbrook [33°44’S 150°27’E], 6 Feb. 1977, T.F. Stanford, 1♂ (AMS KS.1145); Heathcote [34º05’S 151º00’E], 14 Oct. 1973, R. Witchard, 1♀ (AMS KS.5122); Helensburgh [34º12’S 150º59’E], 2020, J. Prangell, 1juv. (ZMH-A0013055); Kingswood [33º46’S 150º43’E], May 1962, 1♀ (AMS KS.4993); Kurrajong [33º33’S 150º40’E], 30 May 1980, M.J. Fletcher, 1♀ (AMS KS.5307); Lakemba [33º56’S 151º05’E], 17 Mar. 1931, P. Bindon, 1♀ (AMS KS.4945); Lane Cove National Park, 33º46’S 151º06’E, 10 Jan. 2020, B. Jones, 1juv. (ZMH-A0012978); Lane Cove National Park [33º47’S 151º09’E], 20 Feb. 2020, B. Buzatto, 1♂ (ZMH-A0012984); Lawson [33°43’S 150°26’E], Mar. 1956, R. Schleicher, 1♂ (AMS KS.1146); Lilli Pilli [34°04’S 151°07’E], Sydney, 16 Jul. 1985, C. Rodwell, 1♂ (AMS KS.17543); Lindfield [33º47’S 151º10’E], 29 Sept. 1962, M. Gregg & V. Gregg, 1♀ (AMS KS.5028); Menai [34º01’S 151º01’E], 10 Aug. 2020, K. Christensen, 1♀ (AMS KS.131641); Mount Wilson [33º31’S 150º22’E], May 1973, P. Kinloch, 1♀ (AMS KS.3605); Mulgoa (Penrith) [33º51’S 150º39’E], Sept. 1993, B. Bone, 1♀ (AMS KS.40766); Penshurst [33º58’S 151º06’E], 1♀ (AMS KS.53217); Pittwater, Jan. 1967, R. Mascord, 1♀ (AMS KS.43798); Potts Point [33°52’S 151°13’E], 5 Jul. 1956, P. Berry Smith, 1♂ (AMS KS.4399); Springwood [33º42’S 150º34’E], 20 Jan. 1956, E. Falcon, 1♀ (AMS KS.1673), 14 Jul. 1971, 1♀ (AMS KS.1217), 1♂ (AMS KS.1138), 3 Oct. 2018, K. Christensen, 1♀ (AMS KS.131642); Stanwell Park [34º14’S 150º59’E], 10 Sept. 1960, R. Newell, 1♀ (AMS KS.5009); Stanwell Tops 2 [Stanwell Tops, 34º13’S 151º00’E], 10 Aug. 2020, K. Christensen, 1♀ (AMS KS.131640); Sutherland [34°02’S 151°03’E], 2 May 1986, M. Mcinnes, 1♂ (AMS KS.16562); Southerland [Sutherland, 34º02’S 151º03’E], 2020, J. Prangell, 1juv. (ZMH-A0013056); Symbio (Helensburgh) [Symbio Wildlife Park, 34º12’S 150º58’E], 7 Aug. 2019, K. Christensen, 1♀ (ZMH-A0012999), 28 Aug. 2019, K. Christensen, 2♀ (ZMH-A0013006, ZMH-A0013032), 29 Oct. 2019, K. Christensen, 1♂ (AMS KS.131647); between Terrigal and Avoca [ca. 33º27’S 151º25’E], 16 Mar. 1981, Kennedy, 1♀ (AMS KS.7235); Warragamba [33º53’S 150º36’E], 17 Jan. 2020, K. Christensen, 1♂ (AMS KS.131643); Windsor [33º37’S 150º49’E], 21 Jan. 2020, K. Christensen, 1♂ (ZMH-A0013005), 23 Apr. 2020, K. Christensen, 1♂ (ZMH-A0013034); Wirrimbirra Sanctuary [34º16’S 150º35’E], nr. Bargo, 9 Jul. 1969, M.N. Fackendet, 1♀ (AMS KS.1848); Woronora Heights [34°02’S 151°02’E], 23 Dec. 1992, 1♂ (AMS KS.37155); Wyong [33º17’S 151º25’E], 19 Jan. 1920, M.E. Piper, 1♀ (AMS KS.16511); Yerranderie [34º07’S 150º13’E], 1978, V. Lhuede, 1♀ (AMS KS.1851).

**Diagnosis.** Adult males are distinguished from all species by the following combination of characters: carapace length x̅ 10.2 (9.0–11.5); palpal tibia 3.3x longer than wide (Fig. 13); embolus 8x longer than wide and with sinuous curvature (Figs. 5B and 17B, D and F). Males can be further distinguished from *A. robustus* by their embolus tip with narrow opening (Fig. 17B, D, F) vs. wider in the latter (Fig. 17A, C, E); from *A. sutherlandi* by their long (8x longer than wide), smoothly curving embolus, while shorter and strongly curved embolus in the latter (see Gray [17]: fig. 12G) and from *A. yorkmainorum* by the slightly twisted embolus with the tip strongly twisted in the latter (see Gray [17]: fig. 10A). Females are distinguished from all species by the combination of the following characters: carapace length x̅ 11.5, (9.1–14.5) and spermathecae (5–6x longer than wide) curved inward; from *A. robustus* which have shorter, straighter spermathecae (4–5x longer than wide), and *A. sutherlandi* which have the spermathecae base wide and touching (3x longer than wide) (see Gray [17]: fig. 13F), while spermathecae base narrow and separated in *Atrax montanus* (Fig. 11).

**Description. Male (AMS KS.131647).** Total length: 21.87; carapace length: 11.18; carapace width: 9.28; abdomen length: 10.69 (Fig. 12). **Colouration** (in ethanol): Carapace, endites and sternum dark brown; chelicerae dark brown; abdomen dark grey; legs reddish-brown. **Carapace**: Broadly oval, pars cephalica slightly elevated; fovea transverse, slightly procurved. **Endites**: With **~** 250 cuspules. **Labium**: With **~** 480 cuspules. **Eyes**: Eye formula 4:4; ocular quadrangle rectangular; AME separated by 1.2x their own diameter; PME separated by 2.8x own diameter. **Sternum**: Longer than wide; three pairs of ovoid sigilla, posterior pair the largest. **Chelicerae**: Rastellum absent; promargin with 14 teeth, retromargin with 16 teeth, furrow with 14 denticles. **Leg measurements**: I 32.56 (8.94/4.54/7.43/8.06/4.49); II 31.31 (8.96/3.66/6.99/7.19/4.51); III 26.86 (7.44/3.01/5.91/6.00/4.50); IV 32.53 (7.69/3.33/7.98/8.08/5.45); leg formula 1423. **Abdomen**: Ovoid; with four spinnerets. **Genitalia**: Palpal tibia 5.57/1.69; bulb 5.17/1.02. Palpal tibia with four macrosetae prolateral; 3.3x longer than wide; embolus 8x longer than wide; sinuous curvature; embolus tip with large, arrow-shaped flap (Figs. 13, 17B, D and F and 18C and D).

**Female Holotype (AMS KS.1039).** Total length: 25.4; carapace length: 11.1; carapace width: 9.4; abdomen length: 12.5 (Fig. 10). **Colouration** (in ethanol): Carapace and legs amber-brown; chelicerae reddish-brown, darker distally; abdomen fawn-brown, slightly mottled; sternum amber-orange with darker margins and orange sigilla; labium and endites orange with black cuspules. **Carapace**: Broadly oval; pars cephalica slightly elevated; fovea transverse, slightly procurved. **Endites**: With ~ 324 cuspules. **Labium**: With ~ 276 cuspules. **Sternum**: Longer than wide; three pairs of ovoid sigilla; posterior pair of sigilla largest, anterior pairs much smaller, subequal. **Chelicerae**: Rastellum absent; promargin with 12 (left) or 14 teeth, retromargin with 13 teeth, furrow with c. 30–35 denticles. **Eyes**: Eye formula 4:4; ocular quadrangle rectangular; AME separated by 1.5x their own diameter; PME separated by 2.0x their own diameter. **Leg measurements**: I 27.7 (8.3/5.0/5.9/5.5/3.0); II 24.8 (7.4/4.3/5.1/4.9/3.1); III 23.1 (6.4/4.0/4.3/4.8/3.6); IV 28.5 (7.5/4.3/5.9/6.6/4.2); leg formula 4123. **Abdomen**: Ovoid; with four spinnerets. **Genitalia**: Internal genitalia with two long spermathecae (5–6x longer than wide), slightly curved inward; oval opening almost touching (Fig. 11).

**Female (AMS KS.131640).** Total length: 23.45; carapace length: 10.41; carapace width: 8.68; abdomen length: 13.04 (Fig. 10). **Colouration** (in ethanol): Carapace and legs reddish-brown; chelicerae dark reddish-brown; abdomen dark grey; sternum orange with darker margins and sigilla; labium and endites dark orange with black cuspules. **Carapace**: Broadly oval; pars cephalica slightly elevated; fovea transverse, slightly procurved. **Endites**: With **~** 305 cuspules. **Labium**: With **~** 344 cuspules. **Sternum**: Longer than wide; three pairs of ovoid sigilla; posterior pair of sigilla largest, anterior pair smallest. **Chelicerae**: Rastellum absent; promargin with 13 teeth, retromargin with 9 teeth, furrow with 24 denticles. **Eyes**: Eye formula 4:4; ocular quadrangle rectangular; AME separated by 1.5x their own diameter; PME separated by 2.5x their own diameter. **Leg measurements**: I 23.97 (6.75/4.03/5.60/4.48/3.11); II 21.84 (6.49/3.29/4.63/4.34/3.09); III 19.27 (4.73/3.46/3.82/4.14/3.12); IV 25.26 (7.23/3.67/5.77/5.47/3.12); leg formula 4123. **Abdomen**: Ovoid; with four spinnerets. **Genitalia**: Internal genitalia with two long spermathecae (5–6x longer than wide), slightly curved inward; oval opening almost touching (Fig. 11).

**Variation.** Male carapace length: 9.0–11.5, x̅ 10.2, *n* = 19; total length: 19.34–25.12, x̅ 22.68, *n* = 5; female carapace length: 9.1–14.5, x̅ 11.5, *n* = 25; total length: 20.99–23.45, x̅ 22.27, *n* = 5. Specimens with male palpal tibia similar to *A. robustus* were noted. Female spermathecae vary in length, width and degree of curvature; some specimens are gently curving, others sharply elbowed; most are strongly convergent distally.

**Distribution.** Found in the southern and northern Sydney Basin bioregion, extending at least as far south as Bowral in the Southern Highlands, and into the upper Blue Mountains to the west (Fig. 3). This species overlaps with *A. robustus* in distribution, however, its range is mainly to the south and west of Sydney.

**Natural History.** Field data suggest that this species is associated with rainforest gullies and requires more shading and moisture than *A. robustus* (Fig. 2A). It builds funnel-webs under rocks or tree logs, often near or in rainforest gullies.

**Taxonomic Remarks.** This species was originally described by Rainbow [10] in the monotypic genus *Poikilomorphia* Rainbow, 1914. It was moved to *Atrax* by Main [15] as *Atrax montanus*, but later synonymised with *A. robustus* by Gray ([16]: p. 114, 115). The spermathecae of the female holotype (AMS KS.1039) from the Blue Mountains is identical to spermathecae of females from Stanwell Tops (AMS KS.131640) and Springwood (ZMH-A0013007) that were sequenced (Fig. 11), but can be easily distinguished from the spermathecae of *A. robustus* based on the characters described above. Considering all available evidence, *A. montanus* is re-validated here.

*Atrax christenseni* Dupérré & Smith sp. nov.

Zoobank: urn: lsid: zoobank.org: pub:54DF0756-7CE4-49A1-B43A-ED1E6C3037AF.

Figures 2B, 3, 4, 5C and F, 14, 15, 16, 17G and H and 18E and F.

**Fig. 14:**
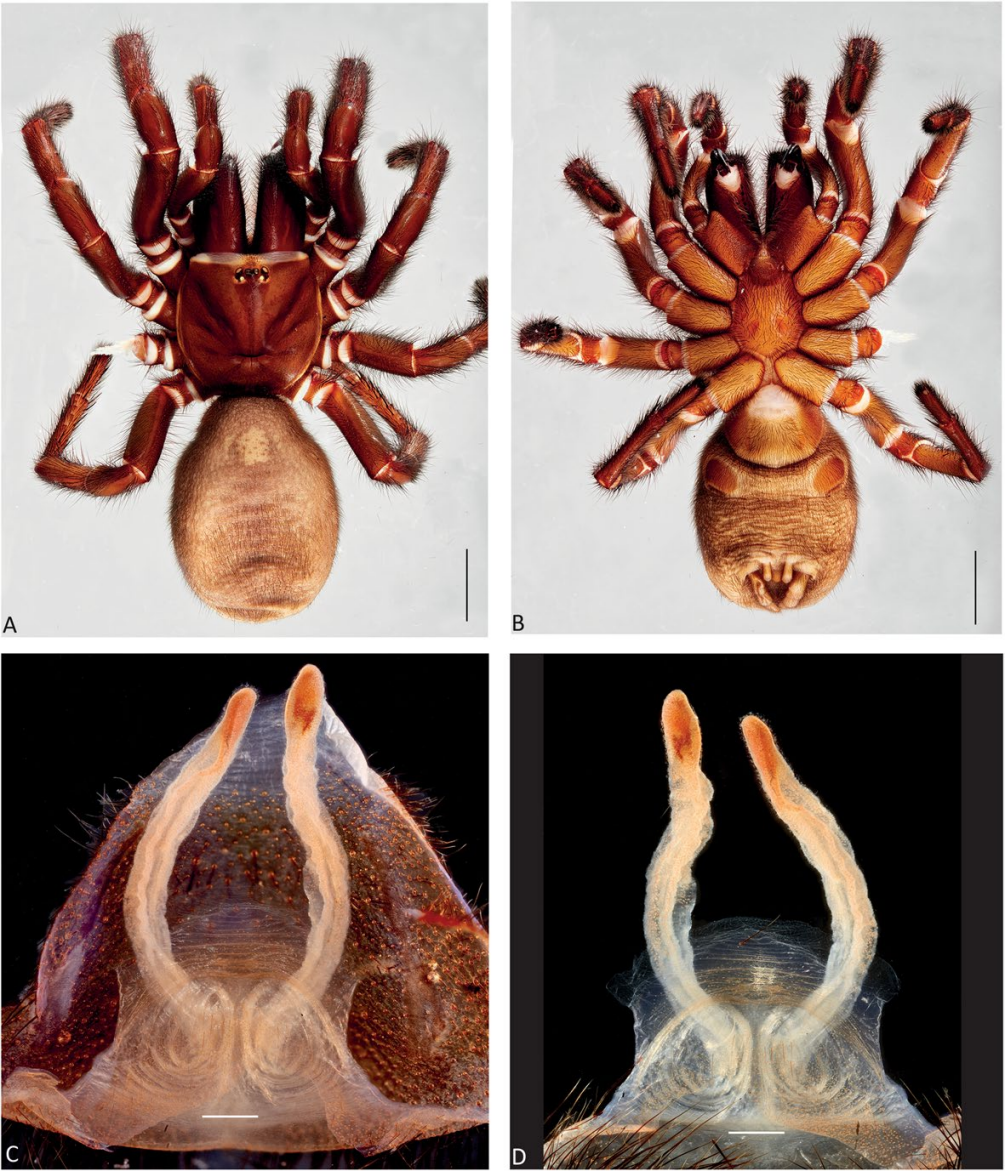
*Atrax christenseni* sp. nov., allotype ♀ (AMS KS.131645). **A**,** B.** Dorsal (**A**) and ventral (**B**) habitus. **C**,** D.** Dorsal (**C**) and ventral (**D**) internal genitalia. Scale bars: **A**,** B** = 5.0 mm; **C**,** D** = 0.5 mm

**Fig. 15:**
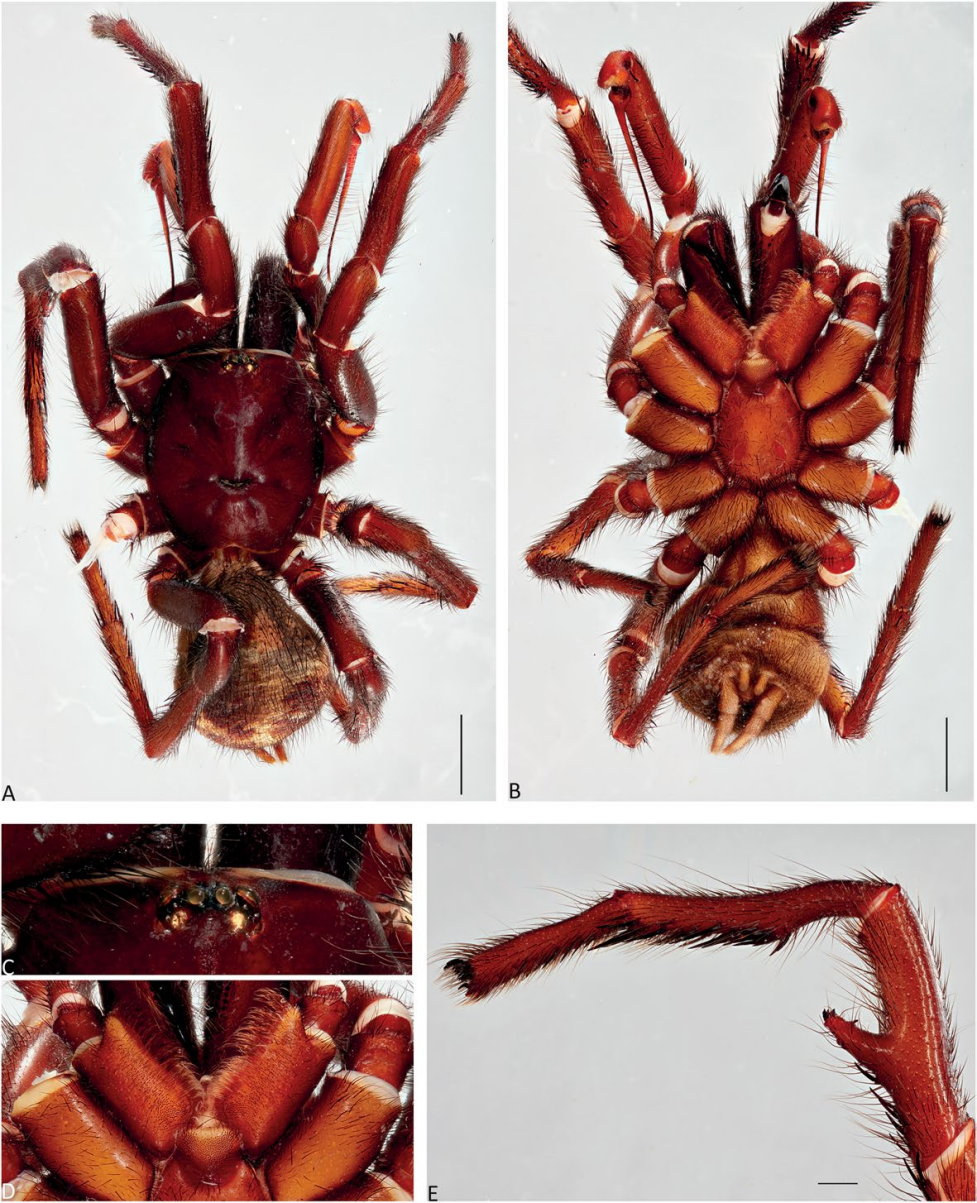
*Atrax christenseni* sp. nov., holotype ♂ (AMS KS.131644), dorsal (**A**) and ventral (**B**) habitus, eyes (**C**), labium and endites (**D**), male leg II (tibia, metatarsus and tarsus), retrolateral view (**E**). Scale bars: **A**,** B** = 5.0 mm; **E** = 1.0 mm

**Fig. 16:**
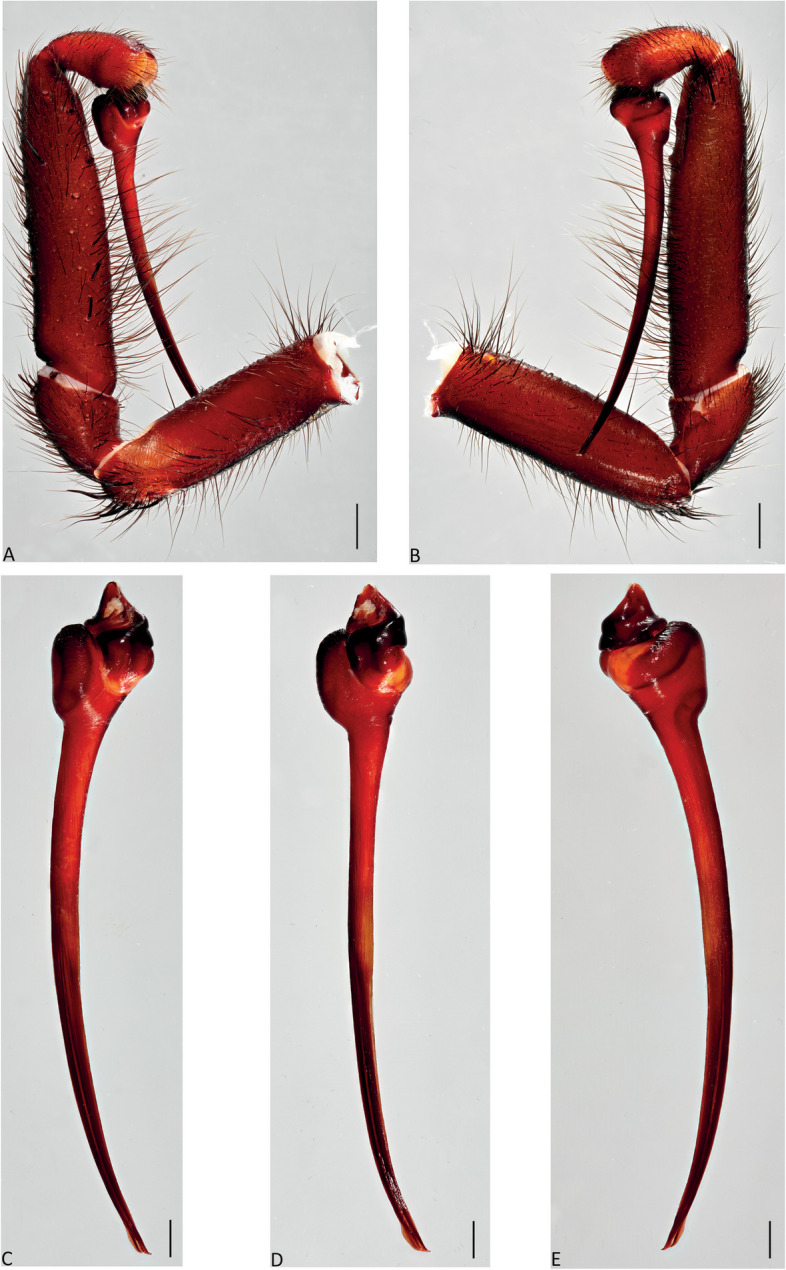
*Atrax christenseni* sp. nov., holotype ♂ (AMS KS.131644). **A**, **B.** Palp, prolateral (**A**) and retrolateral (**B**) views. **C–E.** Bulb and embolus, prolateral (**C**), ventral (**D**) and retrolateral (**E**) views. Scale bars: **A**,** B** = 1.0 mm; **C–E** = 0.5 mm

**Fig. 17:**
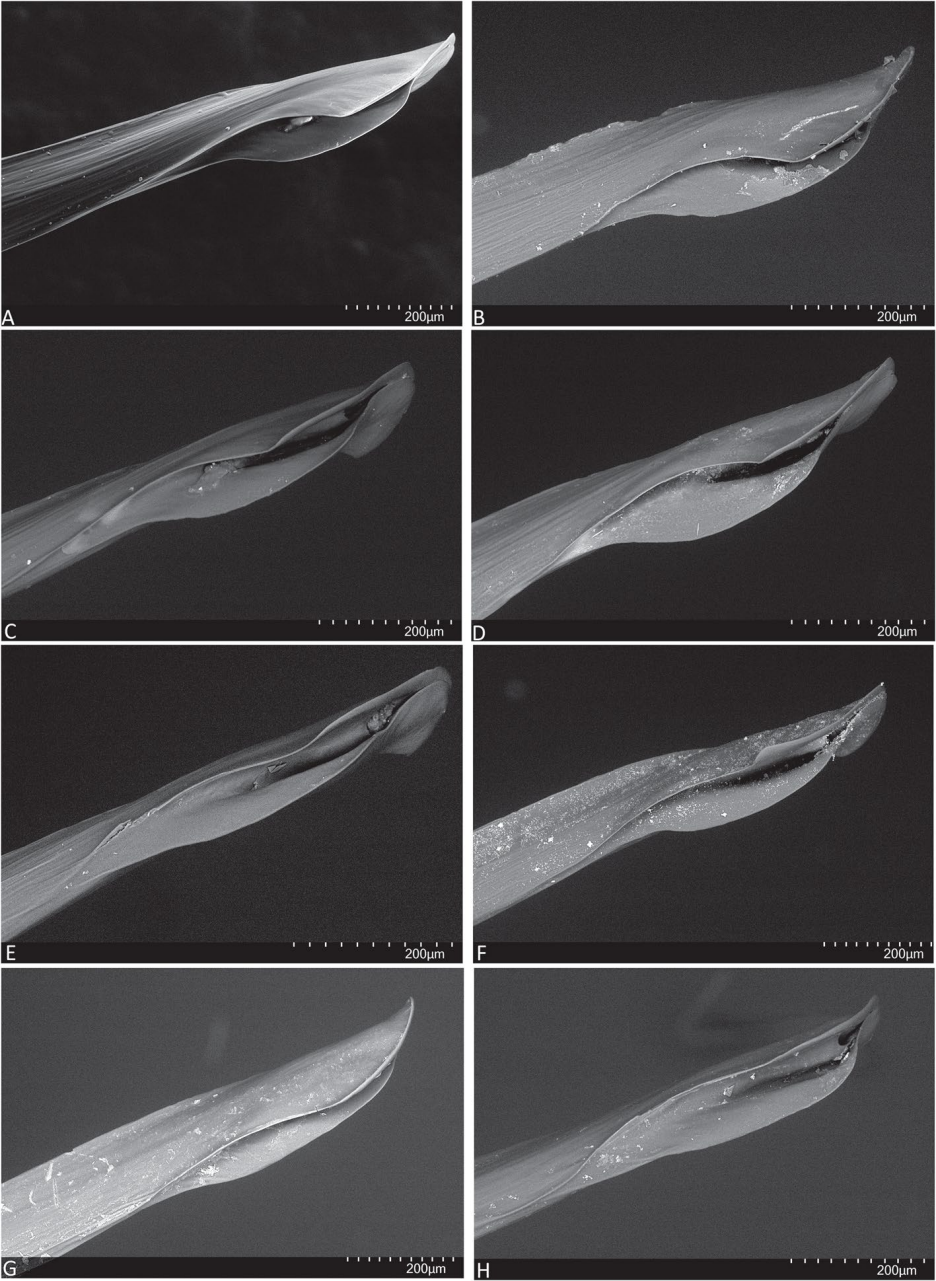
SEM, embolus tip, prolateral (**A**, **B**, **G**) and prolatero-ventral (**C**, **D**, **E**, **F**, **H**) views. **A**, **C**, **E.** *Atrax robustus* O. Pickard-Cambridge, 1877. **B**, **D**, **F.** *Atrax montanus* (Rainbow, 1914). **G**, **H.** *Atrax christenseni* sp. nov.

**Fig. 18:**
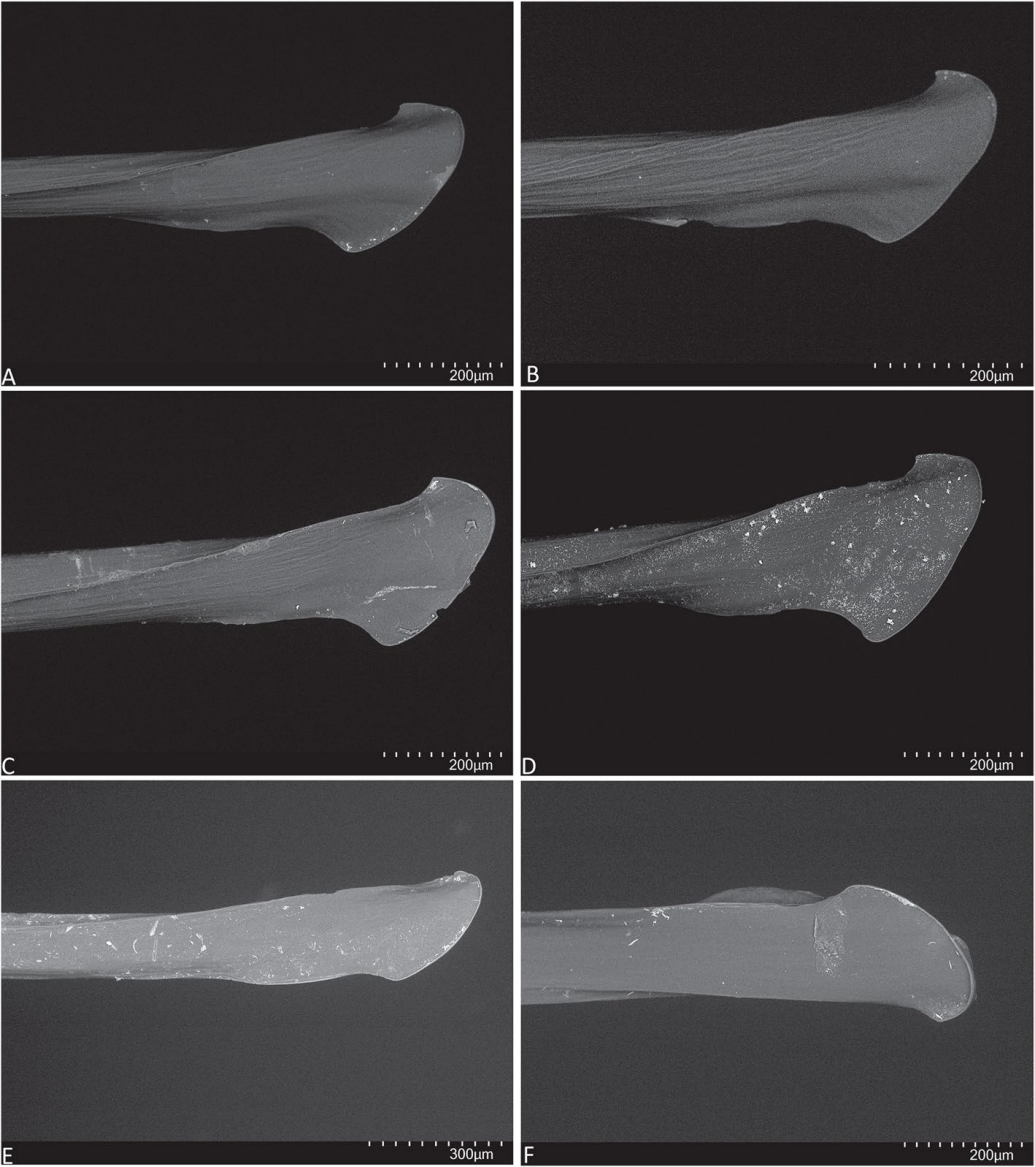
SEM, embolus tip, ventral view. **A**, ** B. ***Atrax robustus* O. Pickard-Cambridge, 1877. **C**, **D.** *Atrax montanus* (Rainbow, 1914). **E**, **F.** *Atrax christenseni* sp. nov.

*Atrax robustus*: Gray [17]: 299, 301 (in part).

**Type Material. AUSTRALIA**: *New South Wales*: all Newcastle area: Holotype ♂ (AMS KS.131644), 13 Feb. 2020, K. Christensen. Allotype ♀ (AMS KS.131645), 8 Jun. 2020, K. Christensen. Paratypes: 1﻿♂ (ZMH-A0012979), 5 Mar. 2009, K. Christensen; 1♂ (ZMH-A0012991), 24 Jan. 2020, K. Christensen; 1♂ (ZMH-A0013020), 6 Feb. 2020, K. Christensen; 1♂ (ZMH-A0013013), 13 Feb. 2020, K. Christensen; 1♂ (AMS KS.131046), 13 Mar. 2020, K. Christensen; 1﻿♂ (ZMH-A0012998), undated, K. Christensen; 2♀ (ZMH-A00024111, AMS KS.131646), 28 Jun. 2022, D. Harms & B.A. Buzatto.

**Material Examined. AUSTRALIA**: *New South Wales*: all Newcastle area: 1♀ (AMS KS.1123), undated, A. Munro; Nov. 1965, J. Splatt, 1♂ (AMS KS.1122); 6 Dec. 1978, R. Mascord, 1♀ (AMS KS.7564); 1979, L. Hallinan, 1﻿♂ (AMS KS.2670); 20 Jan. 1979, R.E. Mascord, 1♀ (AMS KS.4774); 21 Jan. 1979, R.E. Mascord, 1♂ (AMS KS.4773); 22 Jan. 1979, H. Miller, 1♂ (AMS KS.2432); 1 Feb. 1980, G.J. Anderson, 1♂ (AMS KS.4484); 17 Jan. 1982, R. Mascord, 1♂ (AMS KS.10381), 1♀ (AMS KS.10382); 26 Dec. 2004, G. Holl, 1♂ (AMS KS.120552); 3 Feb. 2001, via G. Isbister, 1♂ (AMS KS.120567); 2003, H. Bartlett, 1♂ (AMS KS.88192); 29 Jan. 2020, K. Christensen, 1♂ (AMS KS.131037).

**Etymology.** The specific epithet was chosen in honour of Kane Christensen, whose contributions in collecting spiders were vital to the description of this species.

**Diagnosis.** Adult males are distinguished from all species by their extremely long embolus (12x longer than wide) and widely open embolus tip (Figs. 5C and 17G and H), while shorter in *A. robustus* (6.5x longer than wide), *A. montanus* (8x longer than wide) and *A. sutherlandi* (3.8x longer than wide) (see Gray [17]: fig. 12G); and from *A. yorkmainorum* by the slightly twisted embolus tip (Figs. 5C and 17G and H) that is strongly twisted in the latter (see Gray [17]: fig. 10A). Females differ from all species by their elongate spermathecae (9x longer than wide) that are strongly curved internally and with a large oval entrance almost touching (Fig. 14); *A. robustus* spermathecae 4–5x longer than wide, *A. montanus* 5–6x longer than wide, and *A. sutherlandi* 3x longer than wide (see Gray [17]: fig. 13F).

**Description. Male Holotype (AMS KS.131644).** Total length: 20.88; carapace length: 10.47; carapace width: 8.32; abdomen length: 10.41 (Fig. 15). **Colouration** (in ethanol): Carapace and legs reddish-brown; chelicerae dark reddish-brown; abdomen dark grey; sternum dark reddish-orange with slightly darker margins and sigilla; labium and endites reddish-orange with black cuspules. **Carapace**: Rectangular, pars cephalica slightly elevated; fovea transverse, slightly procurved. **Endites**: With **~** 245 cuspules. Labium: With **~** 404 cuspules. **Sternum**: Longer than wide; three pairs of ovoid sigilla; posterior pair of sigilla largest, anterior pair smallest. **Chelicerae**: Rastellum absent; promargin with 11 teeth, retromargin with 11 teeth, furrow with 14 denticles. **Eyes**: Eye formula 4:4; ocular quadrangle rectangular; ALE separated by 0.7x their own diameter; PME separated by 4x own diameter. **Leg measurements**: I 33.15 (9.82/4.10/7.41/7.33/4.49); II 29.77 (8.86/4.03/6.38/6.60/3.90); III 27.22 (7.20/2.92/5.51/6.33/5.26); IV 32.68 (9.03/3.69/6.95/7.72/5.29); leg formula 1423. **Abdomen**: Ovoid; with four spinnerets. **Genitalia**: Palpal tibia 6.88/1.86; bulb 8.51/1.39. Palpal tibia with four macrosetae prolaterally; 3.7x longer than wide; embolus 12x longer than wide; smooth curvature, embolus tip with small flap and pointed tip (Figs. 16, 17G and H and 18E and F).

**Female Allotype (AMS KS.131645).** Total length: 29.29; carapace length: 12.62; carapace width: 10.37; abdomen length: 17.17 (Fig. 14). **Colouration** (in ethanol): Carapace and legs reddish-brown; chelicerae dark reddish-brown; abdomen light brown; sternum orange with slightly darker margins and sigilla; labium and endites orange-brown with black cuspules. **Carapace**: Rectangular, pars cephalica slightly elevated; fovea transverse, slightly procurved. **Endites**: With **~** 225 cuspules. **Labium**: With **~** 420 cuspules. **Sternum**: Longer than wide; three pairs of ovoid sigilla; posterior pair of sigilla largest, anterior pair smallest. **Chelicerae**: Rastellum absent; promargin with 14 teeth, retromargin with 13 teeth, furrow with 16 denticles. **Eyes**: Eye formula 4:4; ocular quadrangle rectangular; AME separated by 1.4x their own diameter; PME separated by 3.2x their own diameter. **Leg measurements**: I 31.57 (9.92/4.56/7.33/6.32/3.44); II 26.91 (8.31/4.21/5.71/5.51/3.17); III 23.99 (7.25/3.13/5.08/5.24/3.58); IV 28.17 (8.18/3.80/5.94/6.16/4.09); leg formula 1423. **Abdomen**: Ovoid; with four spinnerets. **Genitalia**: Internal genitalia with two long spermathecae (9x longer than wide), slightly curved inward; large oval opening almost touching (Fig. 14).

**Variation.** Male carapace length: 9.7–13.0, x̅ 11.3, *n* = 15; total length: 20.53–24.03, x̅ 21.72, *n* = 4; female carapace length: 11.6–13.5, x̅ 12.4, *n* = 6.

**Distribution.** This species is distributed north of Sydney with all records situated in a 25 km radius around Newcastle (Fig. 3). Exact locations are hidden to protect this species, which occurs across a restricted area and may be endangered by collecting.

**Natural History.** This species seems to build very cryptic funnel-webs (in contrast to *A. robustus*) and this might explain the difficulties in collecting this species over the years (Fig. 2B). Detailed habitat data for recently collected specimens is concealed in the present work to protect this species from overharvesting, but is available for research purposes on the AMS and ZMH collections’ databases. Little else is known but this species is much less common than *A. robustus*. It seems to have a narrow range and is presently endemic to the Newcastle region.

**Taxonomic Remarks.** In his revision, Gray ([17]: p. 301) noted that specimens of *A. robustus* from the Hunter River/Newcastle region were generally larger, and that the pedipalpal tibia and embolus of males was somewhat more elongate. In subsequent years, venom was extracted from several unusually large males collected near Newcastle and the press referred to these males as the “Newcastle big boys”. Based on our molecular phylogeny and the diagnostic characters described above, these specimens near Newcastle represent *A. christenseni*.

## Discussion

### Systematics of *Atrax *in central eastern New South Wales

Our study provides evidence that one of the world’s most iconic spiders is in fact a species complex. The Sydney funnel-web spider sensu Gray, 2010 is divided here into three species that are differentiated both morphologically and phylogenetically, and seem to occupy distinct ranges (with some overlap): *A. robustus* sensu stricto is largely distributed in the wider Sydney metropolitan area and Central Coast with some records from the Blue Mountains and Wollongong; *A. christenseni* sp. nov. is found in the area around Newcastle to the north; and *A. montanus* overlaps with *A. robustus* in the Sydney metropolitan region but extends further west into the upper Blue Mountains, as far south as the Southern Highlands and further north on the Central Coast. Despite the imprecise type locality (New Holland) of *A. robustus*, we confirm that the holotype is morphologically indistinguishable from females now found in the northern suburbs (e.g., Lane Cove National Park) and previously also in south-eastern suburbs – settled soon after the establishment of the British colony – and hence we consider the populations centred around the North Shore of Sydney to represent the real *A. robustus*. Following our revised taxonomy, we recognize five species of *Atrax*: *A. robustus* and *A. montanus* (northern and southern Sydney Basin bioregion, including the Blue Mountains), *A. christenseni* (northern Sydney Basin bioregion in the area around Newcastle), *A. yorkmainorum* (Australian Alps) and *A. sutherlandi* (South Eastern Highlands and coastal regions of south-eastern New South Wales and northeastern Victoria).

Species identification in mygalomorph spiders is a complex endeavour and the general lineage species concept might be applied in such cases to collate different lines of evidence (e.g., phylogenetic, morphological, ecological) to inform taxonomic decisions [55]. The three *Atrax* species examined here share the common attributes of mygalomorph spiders such as population structuring, albeit lower levels likely due to more recent divergence dates: e.g., 1.3–5.8% COI intraspecific divergence for *A. christenseni*, *A. robustus* and *A. montanus*, compared to 22% in the mouse spider species *Missulena davidi* Greenberg et al., 2021 [56]. They also differ morphologically in body size, carapace and ratios of the female (spermathecae) and male (bulb and embolus) genitalia. These differences are consistent, despite some variation noted in species accounts. We also note habitat differences for these species that was observed during our own field work: *A. robustus* is found in open and closed woodlands; *A. montanus* seems to mostly occur in rainforest habitats and is often associated with gullies and drainage lines; and *A christenseni*’s ecology remains poorly understood. Our understanding of *Atrax* ecology and behaviour is still in its infancy [12, 13, 52] and more studies are needed. However, these ecological differences lend further support to our species hypothesis under the general lineage species concept.

It is beyond the scope of this study to taxonomically revise the genus *Atrax* and we stress that the number of funnel-web species might rise in the future. *A. yorkmainorum* and *A. sutherlandi* are currently considered widespread [17], however, detailed studies using wide spatial coverage in combination with molecular phylogenetics might lead to revised species concepts, similar to *A. robustus*. Although *A. yorkmainorum* was excluded from our molecular phylogenetic analysis, it is clearly morphologically distinct from the other *Atrax* species. *A. sutherlandi* females (ZMH-A0016609 and ZMH-A0024181) from Bermagui (the type locality) also possess unique spermathecae, and previous research has shown that this species is genetically structured [19]. We observed a deep divergence between the two *A. sutherlandi* specimens included in our phylogenetic analysis, and incorporating additional samples from across the distribution of *A. sutherlandi*, in combination with detailed morphological work, may also reveal that *A. sutherlandi* is a species complex.

### Biogeography and conservation of *Atrax* in central eastern New South Wales

The genus *Atrax* is too young to be classified as an ancient Gondwana relict and potentially split from *Hadronyche* in the Late Cretaceous [22], as supported by our divergence time estimation analysis (72 Ma). *Atrax* likely began to diversify in the Oligocene (30 Ma), with population divergences occurring as late as Plio-Pleistocene. Vicariant speciation caused by habitat fragmentation induced by Miocene aridification likely contributed to *Atrax* diversification – a common pattern observed in the mesic Australian fauna including mygalomorph spiders [57–64] and other taxa (e.g., [65–69]). However, it is unclear what role other barriers in the Sydney Basin and beyond (e.g., topography of the Blue Mountains, major rivers) played in *Atrax* biogeography, and future research should focus on the species boundaries and identifying factors promoting diversification. What is potentially more interesting is the geographic structuring observed among populations. *Atrax robustus* is comparatively an agile spider with plastic habitat associations. It is often found in disturbed habitats such as gardens and parks (e.g., [1, 70]), and genetic data support the idea of a more continuous distribution in the absence of major population divergences (2.6% COI divergences across all samples). These low divergences could result from relatively recent habitat fragmentation (since the Pliocene), or also indicate a relatively high plasticity in habitat association and dispersal capacity.

Our study also provides a conservation message, even if *Atrax robustus* and its congeners might be seen as pests by many living within the range of these species. Almost all records from metropolitan Sydney (specifically in the area south of Sydney Harbour/Parramatta River and north of Botany Bay/Georges River) in our distribution map (Fig. 3) are pre-2000, yet there are no AMS records of *Atrax* from this area since then. With the rise in digital photography and online identification services, few specimens enter museum collections today, so we cannot comment from AMS records alone. However, iNaturalist has become a useful tool for examining trends in such relatively large and easy to document species. Although *A. robustus* is not listed as threatened or protected by IUCN [71] or CITES [72], our field studies in combination with iNaturalist records and the historical AMS collections suggest a genuine population decline in the more heavily developed Sydney suburbs. In the 1950s, ‘funnel-webs’ were noted to be common in the area of Potts Point, just east of the city centre (AMS KS.4399, *A. montanus*, label data), yet there has been no Australian Museum record from there, or indeed any of the south-eastern suburbs since the 1970s, and there are no reliable recent records in iNaturalist either. Thus *A. robustus* and *A. montanus* may have been almost completely extirpated from this heavily populated area, despite the continuing presence of well vegetated reserves in some steeper gullies. The situation is little better further west, with records since 2000 being only from the Bankstown area. Immediately north of Sydney Harbour, the source of many AMS records of (mostly) *A. robustus*, there are still occasional records on iNaturalist but now fewer than in the more bush-rich areas of the Upper North Shore. Our team’s recent attempts to collect in this area were unsuccessful.

*Atrax christenseni* sp. nov. has a very restricted distribution and is found in remnant bush patches in a small radius surrounding Newcastle and genetic diversity is low. This species might be threatened by urbanization and habitat loss, and possibly even the pet trade, but more research is needed. The same holds true for *A. montanus*, which although widespread, comprises genetically distinct populations that have unique evolutionary potential. The preservation of localized populations in all three species should be prioritized.

### Implications for Venom Research and antivenom production

The present study emphasizes the importance of systematics in an applied framework. Antivenom for *Atrax robustus* was developed in 1981 [73], and since then no fatalities have occurred. Antivenom production relies on wild-caught male specimens, which are dropped off at hospitals by members of the public, and then milked for venom at the Australian Reptile Park. Although this has proven to be an effective approach, some of the variation previously observed in *A. robustus* venom can certainly be attributed to taxonomic confusion, as specimens labeled as *A. robustus* in some venom studies are probably either *A. montanus* or *A. christenseni*. Considering their unique evolutionary histories, it is conceivable that the venom profiles of *A. robustus*, *A. montanus* and *A. christenseni* would differ, and characterizing venom in each of these species may also reveal differences in the presence, quantity and/or structure of delta-atracotoxin – the polypeptide that slows inactivation of sodium-ion channels in primates [74]. In short, the rich body of literature on *A. robustus* venom needs to be re-examined in light of our revised taxonomic framework.

## Conclusions

Antivenom and biomedical research on medically important species ultimately relies on sound taxonomic concepts for the species in question. Here we show that the iconic Sydney funnel-web spider *Atrax robustus* sensu Gray, 2010 is a complex of three species (*A. robustus* sensu stricto, *A. montanus* and *A. christenseni*) that differ phylogenetically and morphologically. Targeted venom analyses of these species might follow, but the findings of past biochemical studies should be re-evaluated in light of a modern taxonomic framework. Antivenom seems to be effective for all *Atrax* species but antivenom specificity to the “real” Sydney funnel-web spider might benefit from acknowledging interspecific boundaries, intraspecific genetic variation, and from considering the distributional range of this species and its congeners. Conservation measures may be warranted to preserve genetic diversity in *Atrax* spp. lineages.

## Supplementary Information

Supplementary Material 1.

## Data Availability

The datasets generated and/or analysed during the current study are available in the Genbank repository (https://www.ncbi.nlm.nih.gov/genbank; see Table S1 for accession codes).
