# Supplementary material for: The world’s most venomous spider is a species complex: systematics of the Sydney funnel-web spider (Atracidae: Atrax robustus)
Source: BMC Ecol Evol. 2025 Jan 13;25:7. doi: 10.1186/s12862-024-02332-0 (PMC11727706; doi:10.1186/s12862-024-02332-0)

**The world’s most venomous spider is a species complex: Systematics of the Sydney funnel-web spider (Atracidae: *Atrax robustus*)**

Stephanie F. Loria^1^, Svea-Celina Frank^1,2^, Nadine Dupérré^1^, Helen M. Smith^3^, Braxton Jones^4^, Bruno A. Buzatto^5,6,^,  Danilo Harms^1,2,3,7*^

^1^ Museum of Nature Hamburg – Zoology, Leibniz Institute for the Analysis of Biodiversity Change, Hamburg, Germany

^2^ Fachbereich Biologie, Universität Hamburg, Hamburg, Germany

^3^ Australian Museum, Sydney, Australia

^4^ School of Life and Environmental Sciences, The University of Sydney, Sydney, Australia

^5^ College of Science and Engineering, Flinders University, Bedford Park, Australia

^6^ Research Adjunct, Western Australian Museum, Welshpool, Australia

^7^ Honorary Research Fellow, Harry Butler Institute, Murdoch University, Murdoch, Australia

* Corresponding author: [d.harms@leibniz-lib.de](mailto:d.harms@leibniz-lib.de)

**Table S1.** GenBank accession codes for DNA sequences of the Domain I region of 28S rDNA (28S), 16S rDNA (16S) and Cytochrome *c* Oxidase Subunit I (COI) loci for phylogenetic analysis of *Atrax robustus* O. Pickard-Cambridge, 1877. Taxa represent the following families Actinopodidae Simon, 1892, Atracidae Hogg, 1901, Atypidae Thorell, 1870, Barychelidae Simon, 1889, Idiopidae Simon, 1889, Nemesiidae Simon, 1889, Pycnothelidae Chamberlin, 1917 and Theraphosidae Thorell, 1869. Tissue samples are deposited at Auburn University Museum (AUM); Australian Museum Sydney, Sydney, Australia (AMS); Museum of Nature Hamburg – Zoology (ZMH), Hamburg, Germany; and Zoological Museum, University of Copenhagen (ZMUC). Specimens originate from the following countries (CO): Australia (AU), Argentina (AR), Denmark (DK), South Africa (SA) and the U.S.A. (US). Asterisk indicates sequences downloaded from GenBank (<https://www.ncbi.nlm.nih.gov/>; Beavis et al., 2011; Wheeler et al., 2017); all other sequences generated in this study.

**Figure S1.** Phylogeny of *Atrax* O. Pickard-Cambridge, 1877 based on Maximum Likelihood analysis of 75 terminals and three genetic markers (COI, 16S and 28S) for a total of 3814 aligned bp, applying the GTRCAT model. Bootstrap and posterior probability values from Maximum Likelihood and Bayesian Inference analyses adjacent to nodes.

**Table S1.** GenBank accession codes for DNA sequences of the Domain I region of 28S rDNA (28S), 16S rDNA (16S) and Cytochrome *c* Oxidase Subunit I (COI) loci for phylogenetic analysis of *Atrax robustus* O. Pickard-Cambridge, 1877. Taxa represent the following families Actinopodidae Simon, 1892, Atracidae Hogg, 1901, Atypidae Thorell, 1870, Barychelidae Simon, 1889, Idiopidae Simon, 1889, Nemesiidae Simon, 1889, Pycnothelidae Chamberlin, 1917 and Theraphosidae Thorell, 1869. Tissue samples are deposited at Auburn University Museum (AUM); Australian Museum Sydney, Sydney, Australia (AMS); Museum of Nature Hamburg – Zoology (ZMH), Hamburg, Germany; and Zoological Museum, University of Copenhagen (ZMUC). Specimens originate from the following countries (CO): Australia (AU), Argentina (AR), Denmark (DK), South Africa (SA) and the U.S.A. (US). Asterisk indicates sequences downloaded from GenBank (<https://www.ncbi.nlm.nih.gov/>; Beavis et al., 2011; Wheeler et al., 2017); all other sequences generated in this study.

| Family | Species | Museum Voucher | CO | Locality | 28S | 16S | COI |
| --- | --- | --- | --- | --- | --- | --- | --- |
| Actinopodidae | *Actinopus* sp. | AUM-MY2873 | AR | Buenos Aires, Parque Leloir | *KY016879 | *KY015695 | *KY017543 |
|  | *Missulena* sp. | AUM-MY2086 | AU | Western Australia | *KY016880 | *KY015696 | *KY017544 |
| Atracidae | *Atrax christenseni* sp. nov. | AMS-KS.131644 | AU |  | PP840698 | PP840639 | PP844782 |
|  |  | AMS-KS.131645 | AU |  | PP840699 | PP840640 | - |
|  |  | ZMH-A0013013 | AU |  | PP840701 | PP840642 | PP844784 |
|  |  | ZMH-A0013020 | AU |  | PP840702 | PP840643 | PP844785 |
|  |  | ZMH-A0012991 | AU |  | PP840700 | PP840641 | PP844783 |
|  | *Atrax montanus* (Rainbow, 1914) | AMS-KS.131640 | AU | Stanwell Tops | PP840703 | PP840644 | PP844786 |
|  |  | AMS-KS.131641 | AU | Menai | PP840704 | PP840645 | PP844787 |
|  |  | AMS-KS.131642 | AU | Springwood | PP840705 | PP840646 | PP844788 |
|  |  | AMS-KS.131643 | AU | Warragamba | PP840706 | PP840647 | PP844789 |
|  |  | AMS-KS.131647 | AU | Symbio (Helensburgh) | - | PP840648 | PP844790 |
|  |  | ZMH-A0012978 | AU | Lane Cove NP | PQ203365 | PQ203368 | PQ203374 |
|  |  | ZMH-A0012984 | AU | Lane Cove NP | PQ203366 | PQ203370 | PQ203376 |
|  |  | ZMH-A0012999 | AU | Symbio (Helensburgh) | PP840707 | PP840649 | PP844791 |
|  |  | ZMH-A0013005 | AU | Windsor | PP840708 | PP840650 | PP844792 |
|  |  | ZMH-A0013006 | AU | Symbio (Helensburgh) | PP840709 | PP840685 | PP844793 |
|  |  | ZMH-A0013032 | AU | Symbio (Helensburgh) | PP840743 | PP840686 | PP844832 |
|  |  | ZMH-A0013034 | AU | Windsor | PP840710 | PP840651 | PP844794 |
|  |  | ZMH-A0013036 | AU | Engadine | PP840711 | PP840652 | PP844795 |
|  |  | ZMH-A0013055 | AU | Helensburgh | PP840712 | PP840653 | PP844796 |
|  |  | ZMH-A0013056 | AU | Southerland [Southerland] | - | PP840687 | PP844833 |
|  |  | ZMH-A0013058 | AU | Belimba Park | - | PP840654 | PP844797 |
|  |  | ZMH-A0013059 | AU | Glenning Valley | PQ203363 | PQ203372 | PQ203378 |
|  | *Atrax robustus* O. Pickard-Cambridge, 1877 | ZMH-A0012976 | AU | Scotland Island | PP840713 | PP840655 | PP844798 |
|  |  | ZMH-A0012977 | AU | Deep Creek Reserve | PP840714 | PP840656 | PP844799 |
|  |  | ZMH-A0012980 |  | Ku-ring-gai Chase NP | - | PQ203369 | PQ203375 |
|  |  | ZMH-A0012983 | AU | Scotland Island | PP840715 | PP840657 | PP844800 |
|  |  | ZMH-A0012985 | AU | via Hornsby hospital | PP840744 | PP840688 | PP844834 |
|  |  | ZMH-A0012986 | AU | Central Coast area | PP840716 | PP840658 | PP844801 |
|  |  | ZMH-A0012988 | AU | Cook St Trail | - | PP840659 | PP844802 |
|  |  | ZMH-A0012997 | AU | Ku-ring-gai Chase NP | PP840717 | PP840660 | PP844803 |
|  |  | ZMH-A0013001 | AU | Gosford | PP840718 | PP840661 | PP844804 |
|  |  | ZMH-A0013002 | AU | Matcham | PP840719 | PP840662 | PP844805 |
|  |  | ZMH-A0013004 | AU | Hornsby Hospital | PP840720 | PP840663 | PP844806 |
|  |  | ZMH-A0013009 | AU | Hornsby Hospital | PP840721 | PP840664 | PP844807 |
|  |  | ZMH-A0013010 | AU | Hornsby Hospital | - | PP840665 | PP844808 |
|  |  | ZMH-A0013011 | AU | Asquith | - | PP840666 | PP844809 |
|  |  | ZMH-A0013012 | AU | Bilgoa Plateau | PP840722 | PP840667 | PP844810 |
|  |  | ZMH-A0013014 | AU | Terrey Hills | PP840723 | PP840668 | PP844811 |
|  |  | ZMH-A0013015 | AU | Brookvale | PP840724 | PP840669 | PP844812 |
|  |  | ZMH-A0013016 | AU | Galston | - | PQ203371 | PQ203377 |
|  |  | ZMH-A0013018 | AU | Terrey Hills | PP840725 | PP840670 | PP844813 |
|  |  | ZMH-A0013019 | AU | Beecroft | PP840726 | PP840671 | PP844814 |
|  |  | ZMH-A0013021 | AU | Beecroft | PP840727 | PP840672 | PP844815 |
|  |  | ZMH-A0013030 | AU | Mona Vale Vet | PP840728 | PP840673 | PP844816 |
|  |  | ZMH-A0013037 | AU | Hornsby | PP840729 | PP840689 | PP844817 |
|  |  | ZMH-A0013038 | AU | Terrigal | PP840730 | PP840674 | PP844818 |
|  |  | ZMH-A0013042 | AU | Deep Creek Reserve | - | PP840675 | PP844819 |
|  |  | ZMH-A0013043 | AU | Bidjigal Reserve [Bidigal Reserve] | PP840731 | PP840676 | - |
|  |  | ZMH-A0013047 | AU | Garigal NP | PP840732 | PP840677 | PP844820 |
|  |  | ZMH-A0013049 | AU | Garigal NP | PP840733 | PP840678 | PP844821 |
|  |  | ZMH-A0013050 | AU | Lane Cove NP | PP840745 | PP840690 | PP844835 |
|  |  | ZMH-A0013052 | AU | Cook St Trail | PP840734 | PP840679 | PP844822 |
|  |  | ZMH-A0013053 | AU | Cumberland State Forest | PP840746 | PP840691 | PP844836 |
|  |  | ZMH-A0013057 | AU | Wyoming Vet | - | PP840680 | PP844823 |
|  |  | ZMH-A0013061 | AU | Bilpin | PQ203364 | PQ203373 | PQ203379 |
|  |  | ZMH-A0013062 | AU | Berowra Valley NP, Benowie Walking Track | PP840735 | PP840681 | PP844824 |
|  | *Atrax sutherlandi* Gray, 2010 | OA49 | AU | Tallaganda | - | - | *JN130170 |
|  |  | OQ07 | AU | Great Dividing Range | - | - | *JN130191 |
|  |  | ZMH-A0013063 | AU | West Pennant Hills | PP840736 | PP840682 | PP844825 |
|  | *Hadronyche* *cerberea* L. Koch, 1873 | ZMH-A0012990 | AU | Umina Beach | PP840741 | PP840696 | PP844831 |
|  |  | ZMH-A0013027 | AU | Bucketty | PP840737 | PP840692 | PP844826 |
|  | *Hadronyche formidabilis* (Rainbow, 2014) | ZMH-A0012981 | AU | Barrington Tops | - | PP840695 | PP844837 |
|  |  | ZMH-A0012992 | AU | Watagans | PP840738 | PP840683 | PP844827 |
|  |  | ZMH-A0013060 | AU | Berowra Valley NP | PP840742 | PP840697 | - |
|  | *Hadronyche levittgreggae* Gray, 2010 | ZMH-A0013023 | AU | Kellyville | - | PP840693 | PP844828 |
|  | *Hadronyche lynabrae* Gray, 2010 | ZMH-A0012995 | AU | Tumbi Umbi | PP840739 | PP840684 | PP844829 |
|  | *Hadronyche mascordi* Gray, 2010 | ZMH-A0012993 | AU | Central Mangrove | PP840740 | PP840694 | PP844830 |
| Atypidae | *Atypus affinis* Eichwald, 1830 | ZMUC | DK | Zealand, NWZ, Roesnaes, VindekildeS of Roesnaesgaarden | *KY016939 | *KY015750 | *KY017595 |
| Barychelidae | *Synothele arrakis* Raven, 1994 | AUM-MY2135 | AU | 38 km N of Menzies | *KY016947 | *KY015758 | *KY017602 |
| Idiopidae | *Idiosoma manstridgei* (Pocock, 1897) | AUM-MY2152 | AU | Korrelocking | *KY017133 | *KY015914 | *KY017757 |
| Nemesiidae | Nemesiidae sp. | AUM-MY551 | SA | KwaZulu-Natal, Ngome Forest | *KY017215 | *KY015984 | *KY017809 |
| Pycnothelidae | *Stanwellia hoggi* (Rainbow, 1914) | AUM-MY2092 | AU | ~2 km N of Stanwell Park | *KY017216 | *KY015985 | *KY017810 |
| Theraphosidae | *Aphonopelma vorhiesi* (Chamberlin & Ivie, 1939) | AUM-MY2464 | US | New Mexico, nr. “Las Cruces” | *KY017408 | *KY016156 | *KY017963 |

**References**

Beavis AS, Sunnucks P, Rowell DM. Microhabitat preferences drive phylogeographic disparities in two Australian funnel web spiders. Biological Journal of the Linnean Society. 2011;104(4):805–19.<https://doi.org/10.1111/j.1095-8312.2011.01753.x>

Wheeler WC et al. The spider tree of life: Phylogeny of Araneae based on target‐gene analyses from an extensive taxon sampling. Cladistics. 2017;33(6):574–616.<https://doi.org/10.1111/cla.12182>

**Figure S1.** Phylogeny of *Atrax* O. Pickard-Cambridge, 1877 based on Maximum Likelihood analysis of 75 terminals and three genetic markers (COI, 16S and 28S) for a total of 3814 aligned bp, applying the GTRCAT model. Bootstrap and posterior probability values from Maximum Likelihood and Bayesian Inference analyses adjacent to nodes.


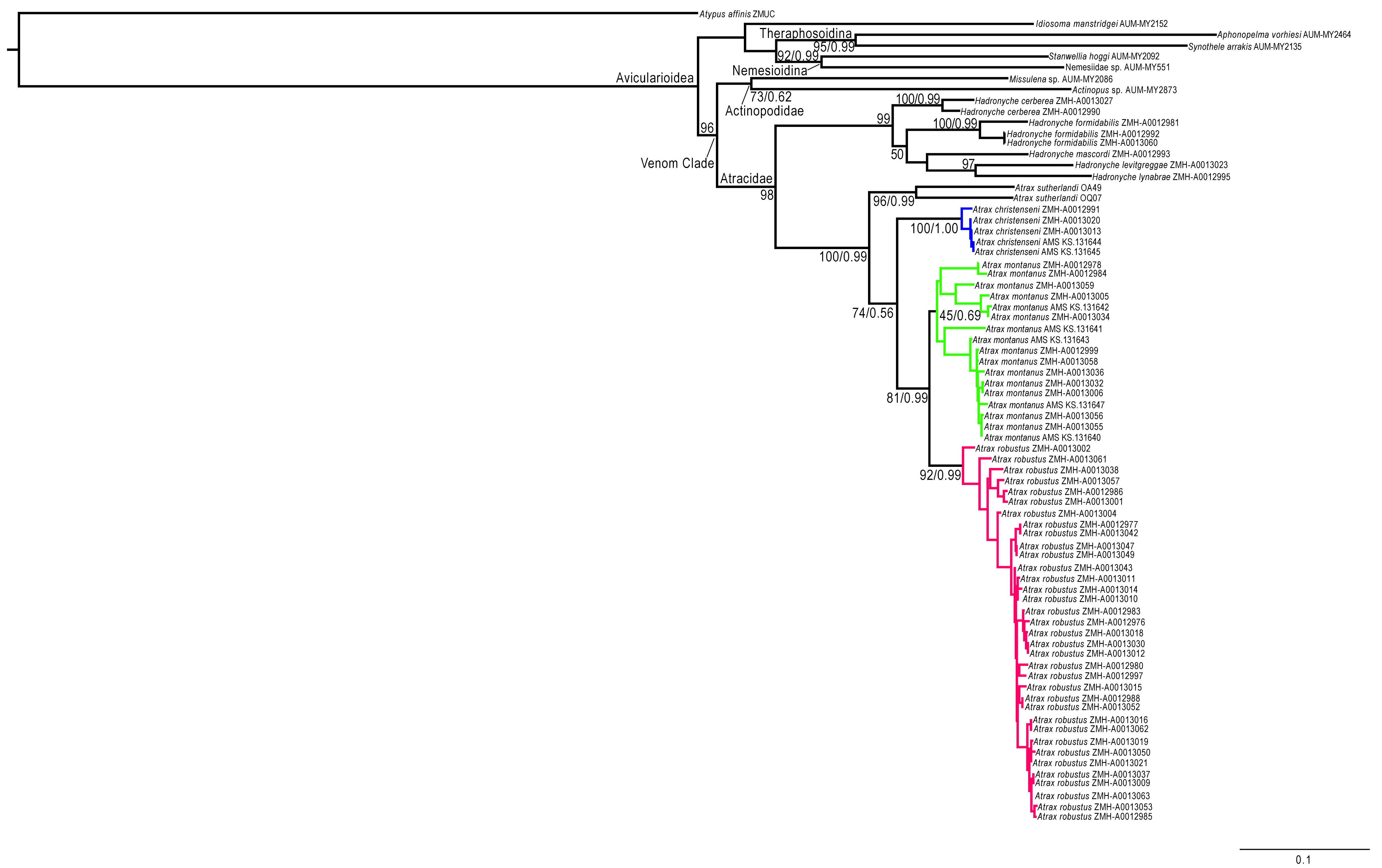

Supplement: Supplementary file 1 — Supplementary Material 1. [file 12862_2024_2332_MOESM1_ESM.docx]
